# Mitochondrial Fusion and Fission in Age‐Related Cardio‐Cerebral Diseases: Mechanisms and Interventions

**DOI:** 10.1111/acel.70729

**Published:** 2026-09-23

**Authors:** Yuyao Yin, Zijian Li

**Affiliations:** ^1^ Department of Cardiology Shengjing Hospital of China Medical University Shenyang Liaoning China; ^2^ Department of Neurology Shengjing Hospital of China Medical University Shenyang Liaoning China

**Keywords:** age‐related disease, cardiovascular disease, fission and fusion, mitochondrial dynamics, neurodegenerative disease

## Abstract

Mitochondria play a crucial role in cellular energy metabolism. The heart and brain require a continuous and stable energy supply. Energy production strongly depends on proper mitochondrial function. Mitochondrial fusion and fission, known as “plasticity”, are vital for maintaining the normal physiological function of cells. Recent studies have shown that impaired mitochondrial dynamics are present in many aging‐related diseases, including Alzheimer's disease (AD), Parkinson's disease (PD), and atherosclerotic cardio‐cerebrovascular disease. The role of impaired mitochondrial dynamics in the pathophysiological process of aging‐related diseases is being actively researched. We discovered that targeting proteins related to mitochondrial dynamics, especially those involved in fission and fusion, may offer new treatment strategies for these diseases. Various approaches, including aerobic interval and treadmill training and the use of drugs such as the antidiabetic agents metformin and dapagliflozin, the antihypertensive agent irbesartan, and certain traditional Chinese medicine components, have shown potential in alleviating imbalances in mitochondrial dynamics in aging‐related cardio‐cerebrovascular diseases. In this review, we systematically summarize recent research on alterations in mitochondrial dynamics in age‐related cardio‐cerebral diseases and explore therapeutic strategies targeting these alterations, which may offer new directions for improving cardiac and brain health and guiding clinical practice.

## Graphical abstract

Mitochondrial fission‐fusion imbalance contributes to age‐related cardio‐cerebral diseases like Alzheimer's disease, Parkinson's disease, Cerebral Infarction, Arteriosclerosis, Myocardial Infarction, Cardiac valve disease, Hypertension, and Cardiomyopathy. This review summarizes its regulatory mechanisms and interventions. (The graphical abstract was created with Figdraw).

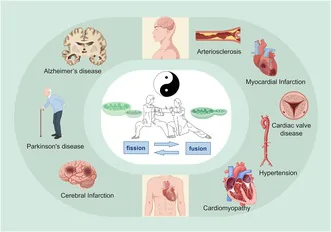

## 1 Introduction

**FIGURE 1:**
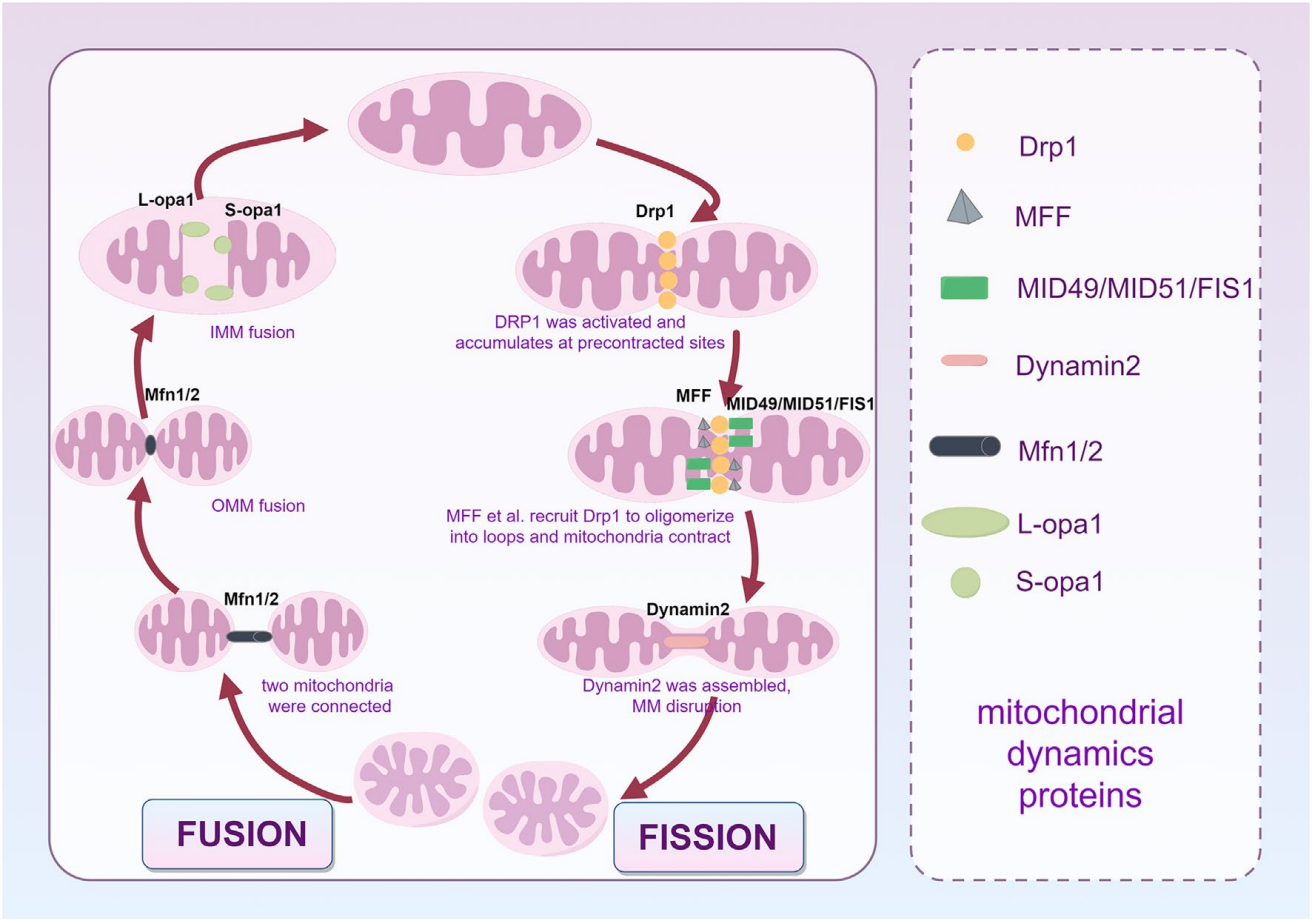
Schematic illustration of mitochondrial dynamics: molecular mechanisms of fusion and fission (by Figdraw).

The disruption of mitochondrial homeostasis has been implicated in various pathological processes, including the onset and progression of hereditary, degenerative, and metabolic diseases and cancer. Maintaining this homeostasis is crucial for cellular survival and organ physiological function. As core organelles that regulate cellular energy metabolism and intracellular signaling, mitochondria are indispensable for sustaining the physiological functions of high‐energy‐consuming organs such as the heart and brain. The heart relies on a continuous supply of mitochondrial energy to sustain the contraction–relaxation cycle of cardiomyocytes, whereas neurons in the brain depend on mitochondria to ensure the transmission of neural impulses and the maintenance of synaptic function under high metabolic states. Mitochondrial dynamics refer to the dynamic equilibrium of the morphology and number of mitochondria. As mitochondria are the core organelles associated with cellular energy metabolism, this equilibrium is vital for maintaining cellular function and homeostasis (Kondadi and Reichert 2024). Mitochondrial dynamics primarily regulate fusion and fission processes, which play key roles in maintaining mitochondrial network homeostasis and ensuring a continuous supply of ATP. Fusion promotes the formation of larger mitochondrial structures, increasing the efficiency of energy production, whereas fission distributes energy substrates to more mitochondria, allowing them to adapt to the varying energy demands of cardiomyocytes under different physiological states (Mendelsohn et al. 2022). Similarly, through the coordinated regulation of fusion‐ and fission‐related proteins, neurons achieve a stable energy supply to meet their high metabolic requirements (Simões et al. 2021). Proteins related to mitochondrial dynamics belong to the dynamin family and possess GTPase activity to modulate mitochondrial size, morphology, and distribution according to cellular physiological demands. In mammals, key fusion proteins include optic atrophy‐related protein 1 (Opa1) and mitochondrial fusion proteins 1/2 (Mfn1/2). Opa1 primarily governs inner mitochondrial membrane (IMM) fusion, whereas Mfn1 and Mfn2 mediate outer mitochondrial membrane (OMM) fusion. Mitochondrial fission is driven primarily by membrane constriction mediated by dynamin‐related protein 1 (Drp1) (Chan 2020) and membrane cleavage facilitated by dynamin‐related protein 2 (Dnm2) (Lee et al. 2016). This fission process also requires the synergistic action of Drp1 adaptor proteins such as Mff, Mid49, Mid51, and Fis1 (Atkins et al. 2016). Posttranslational modifications (phosphorylation, S‐nitrosylation, SUMOylation, and acetylation) and liquid–liquid phase separation act as molecular switches that regulate core dynamic proteins, thereby disrupting mitochondrial network homeostasis without altering protein expression. Recent advances in cryo‐electron microscopy have revealed the complex molecular mechanisms underlying the assembly of fusion and fission proteins, providing atomic‐level structural insights for the development of conformation‐selective small‐molecule modulators (Franco et al. 2016). Consequently, the development of small‐molecule drugs targeting mitochondrial dynamics‐related proteins has become highly promising in translational medicine (Figure 1).

Mitochondrial dysfunction ranks among the ten hallmarks of aging and plays a pivotal role in the pathogenesis of cardiac and neurodegenerative diseases. During aging, abnormal changes in mitochondrial morphology and function exacerbate oxidative stress, leading to mitochondrial DNA (mtDNA) degradation, triggering apoptosis, and accelerating the aging process. Furthermore, aging disrupts mitochondrial dynamic equilibrium. Studies involving aging models such as yeast, *Drosophila*, and *Caenorhabditis elegans* have indicated that dynamic changes in mitochondrial fusion and fission significantly influence organismal lifespan (Rana et al. 2017; Scheckhuber et al. 2007). The modulation of mitochondrial dynamics extends the lifespan of model organisms. However, human cohort studies are needed to establish a causal relationship between mitochondrial dynamics and age‐related cardio‐cerebral diseases and to assess heterogeneity across sexes and ethnicities. Multiple investigations have confirmed a close association between imbalances in mitochondrial dynamics and age‐related diseases, yet the causal relationship between mitochondrial dynamics and human lifespan remains unclear. Recent studies have summarized the effects of mitochondrial dysfunction on cardiovascular and neurological diseases (Gallo et al. 2024; Zhang, Zhu, et al. 2024). However, systematic investigations that focus on the dysregulation of mitochondrial dynamics as a shared core mechanism in patients with cardio‐cerebral comorbidities remain limited.

**FIGURE 2:**
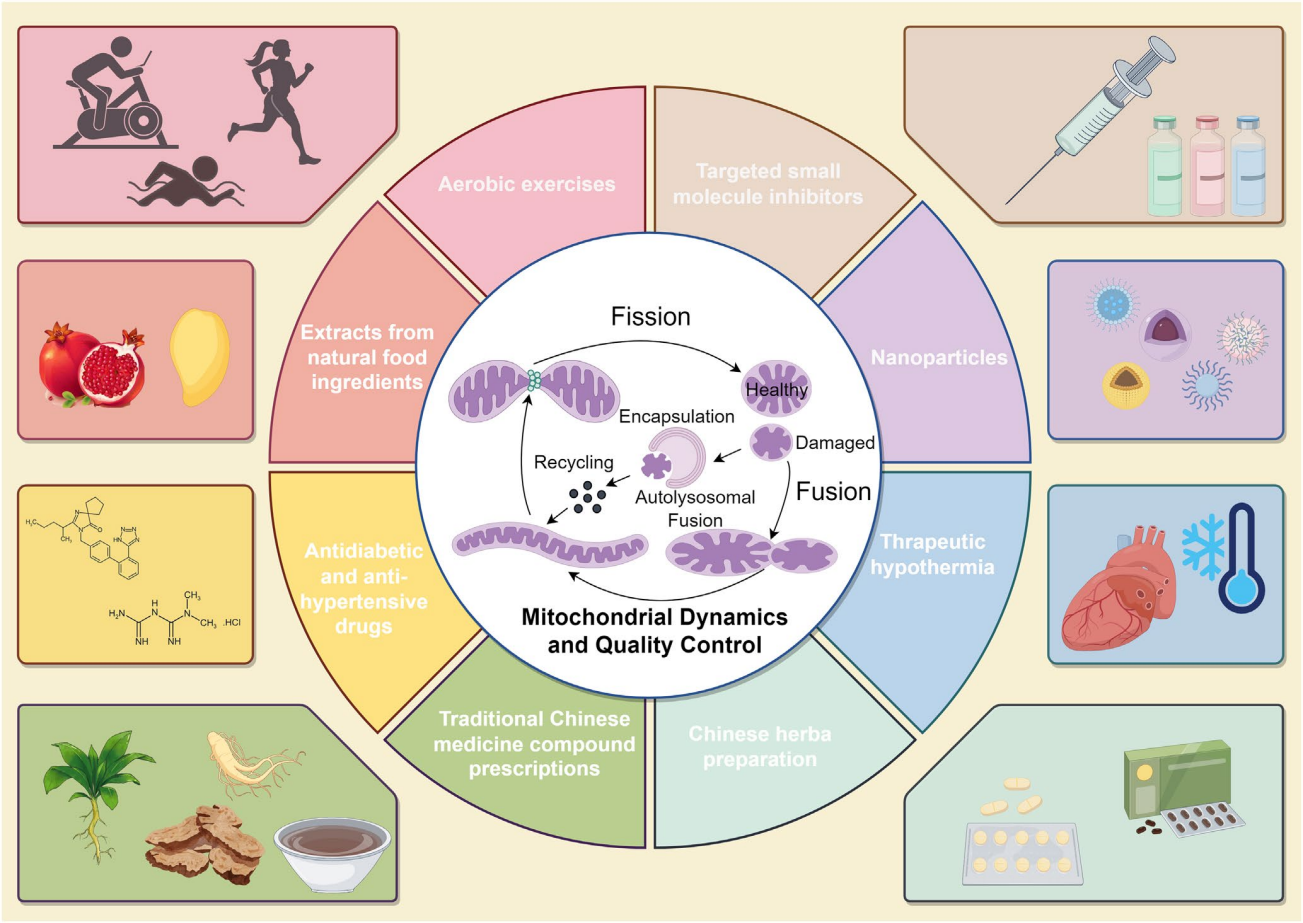
Therapeutic strategies targeting mitochondrial dynamics for age‐related cardio‐cerebral diseases (by Figdraw).

With the accelerating aging of the global population, the comorbidity of degenerative heart and brain diseases among elderly people has become widespread, posing a major challenge to healthcare systems worldwide. In this review, the dysregulation of mitochondrial dynamics observed in cardiac and neurodegenerative diseases is systematically examined, how mitochondrial dysfunction, a shared molecular mechanism, regulates distinct pathological processes across these conditions is explored, and therapeutic strategies targeting mitochondria‐related proteins are summarized. Recognizing mitochondrial dysfunction as a common precipitating factor for cardiovascular and neurological diseases facilitates the development of effective integrated treatment approaches. This review synthesizes existing research on the disruption of mitochondrial dynamics in cardiac and brain disorders. We assess common and organ‐specific regulatory mechanisms, identify key unresolved issues, and discuss potential therapeutic strategies for age‐related cardiovascular and cerebrovascular diseases. These efforts aim to inform future anti‐aging research and ultimately improve human health (Figure 2).

## 2 Structural Composition and Functional Compartmentalization of Mitochondria

Mitochondria are double‐membrane organelles that are composed of four main structures: the OMM, the intermembrane space (IMS), the IMM, and the matrix. Each structure functions independently but coordinates with the other structures to collectively perform the basic physiological functions of mitochondrial energy production and maintenance of cellular homeostasis. The OMM consists of a phospholipid bilayer, with phospholipids accounting for two‐thirds of the total membrane lipids, which separates the mitochondria from the cytoplasmic environment (Pemberton et al. 2025). Voltage‐dependent anion channels (VDACs) regulate the permeability of the outer membrane and are responsible for the transport of various metabolic substances, such as ATP and ADP. Additionally, the OMM is associated with core protein complexes involved in mitochondrial fusion and fission, which participate in the regulation of mitochondrial morphological changes (de Pinto et al. 2022). The OMM has relatively high permeability, whereas the IMM folds inward to form cristae, effectively increasing the surface area for oxidative phosphorylation (OXPHOS) (Wolf et al. 2019). The IMM is the primary site for ATP synthesis in cells; complexes I–IV of the electron transport chain (ETC) are distributed across the membrane and transfer electrons from NADH, FADH_2_, and cytochrome c to oxygen to complete the energy conversion process (Minjares et al. 2023). Mitochondrial calcium uniporters (MCUs) and Na^+^/Ca^2+^ exchangers (NCXs) on the IMM help maintain stable intracellular calcium levels (Takeuchi and Matsuoka 2021). Concurrently, enzymes distributed on the inner membrane, such as superoxide dismutase (SOD) and glutathione peroxidase (GPX), regulate the levels of reactive oxygen species (ROS) to maintain normal mitochondrial morphological functions. The IMS is the smallest structural component of the mitochondria. It maintains normal mitochondrial physiological states by regulating protein transport, folding, and cellular signaling. The IMS possesses specific protein transport pathways capable of transporting key proteins required for maintaining mtDNA stability and regulating cell death and protein folding (Weith et al. 2025). These proteins are first sorted and transported through the OMM; upon entering the IMS, they are assembled via cysteine‐mediated disulfide bond formation pathways (Mesecke et al. 2005). The IMS participates in cellular signaling by regulating calcium ion signaling and ROS production; dysfunction of this structure can result in mitochondrial damage, leading to metabolic disorders, immune dysfunction, and neurological diseases (Goyani et al. 2024). The matrix is a liquid structure enclosed by the IMM and contains metabolic substrates, functional enzymes, mtDNA, and ribosomes (Mishra and Chan 2016). It serves as the site of the tricarboxylic acid (TCA) cycle and participates in regulating mitochondrial morphological changes. Carbohydrates, lipids, and proteins undergo catabolism via the TCA cycle within the matrix; the resulting reduced substances activate the ETC within the inner membrane, resulting in the generation of a transmembrane proton gradient, which ultimately leads to ATP synthesis via F_1_F_0_‐ATP synthase. The TCA cycle produces GTP, which serves as a substrate for GTPases such as mitochondrial fusion proteins 1 and 2 (MFN1/2) and Drp1 (Mishra and Chan 2016). Decreased TCA cycle activity leads to insufficient GTP synthesis, inhibiting the functions of MFN1/2 and Drp1 and hindering mitochondrial structural remodeling. Additionally, the matrix maintains mitochondrial morphological stability through three mechanisms: metabolite transport, assistance in protein folding, and degradation of abnormal proteins. The regulatory mechanisms of various mitochondrial structures function together with the IMM structure and ETC complexes. While ensuring energy production, mitochondria can adjust their morphology in response to cellular calcium signals and metabolic states, making them crucial organelles involved in metabolic regulation, structural maintenance, and signal transduction within the cell.

## 3 Mitochondrial Dynamics: Molecular Regulation of Fusion and Fission and Pathophysiological Implications

**FIGURE 3:**
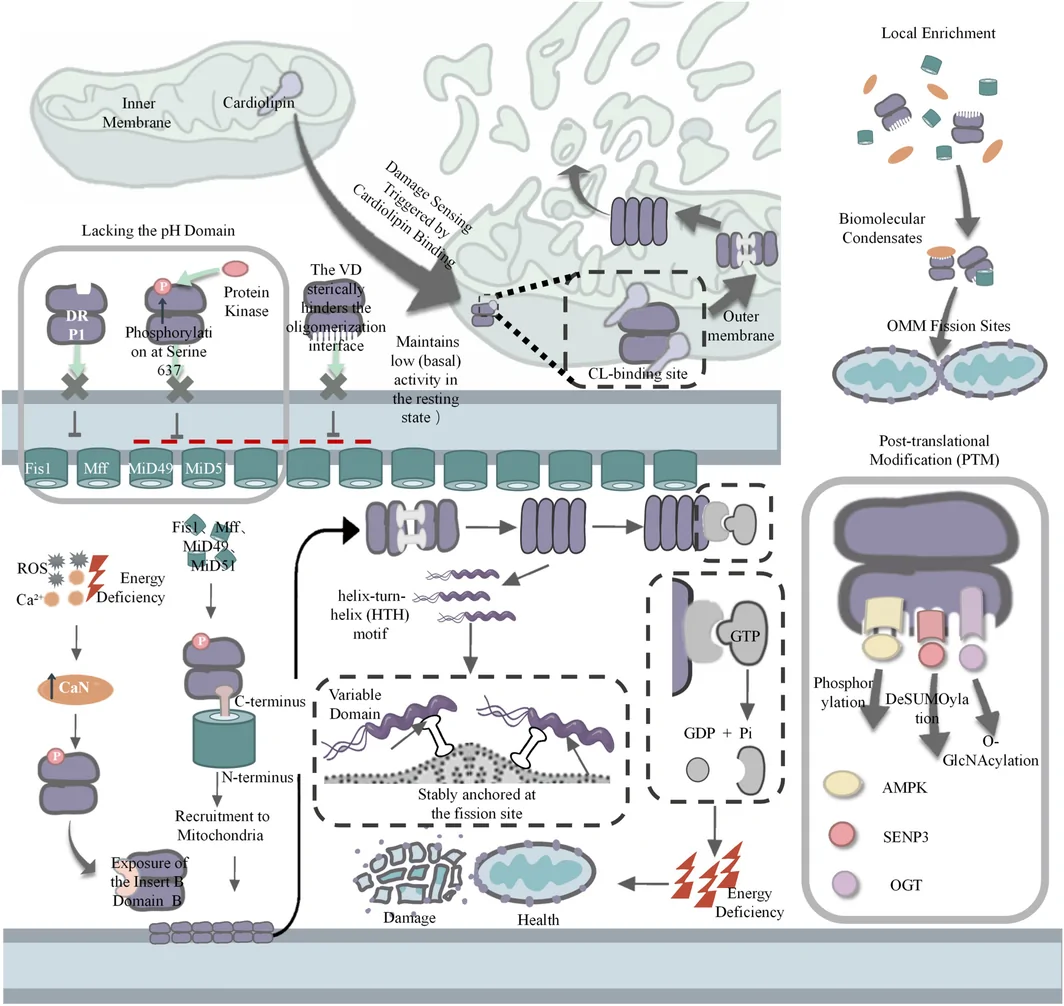
Schematic illustration of mitochondrial dynamics: fusion, fission, and regulatory mechanisms in health and disease. This schematic outlines the core mechanisms of mitochondrial dynamics, where mitochondrial fusion is mediated by mitofusins and OPA1 to repair damaged components and maintain genetic integrity, while fission is driven by Drp1 recruited to the OMM via receptors, with Drp1's VD integrating signals through conformational changes, post‐translational modifications (phosphorylation, SUMOylation), and liquid–liquid phase separation to enable oligomerization and ring contraction for mitochondrial division; this dynamic balance between fusion and fission sustains mitochondrial function, and its dysregulation, such as MFN2 mutations or excessive Drp1 activation, contributes to mitochondrial dysfunction, linking to aging and diseases like ADOA and neurodegenerative/cardiovascular disorders.

Imbalances in mitochondrial fusion and fission are key hallmarks of cellular senescence; a dynamic equilibrium between these two processes enables the maintenance of mitochondrial morphology and metabolic stability and protects the genome from damage (Camacho‐Encina et al. 2024; Marei 2026). This process is regulated primarily by the functional state and posttranslational modifications of core GTPases rather than by protein expression levels (Wu and Mao 2026). Mitochondrial outer membrane fusion is mediated by two proteins, MFN1 and MFN2. Although these two proteins have similar functions, their roles differ. MFN1 primarily accelerates the membrane fusion process (Alghamdi 2024). In addition to participating in outer membrane fusion, MFN2 maintains the structural connection between mitochondria and the endoplasmic reticulum (ER), regulates cellular calcium signaling and lipid transport, and provides the fundamental conditions for outer membrane fusion (Alsayyah et al. 2024; Hong et al. 2025; Kumar et al. 2024). In cells lacking MFN2, calcium homeostasis is disrupted; simultaneous knockout of both MFN1 and MFN2 completely blocks mitochondrial outer membrane fusion, leading to abnormal assembly of the mitochondrial respiratory chain (Chen, Zhao, and Li 2023; Fan et al. 2020). Mitochondrial inner membrane fusion is enabled by the OPA1 protein. The proteases OMA1 and YME1L regulate the ratio of long‐chain OPA1 (L‐OPA1) to short‐chain OPA1 (S‐OPA1) (Ahola et al. 2024; Daumke and van der Laan 2025). Under normal physiological conditions, both OPA1 subtypes work together to maintain the intact structure of the mitochondrial inner membrane cristae (von der Malsburg et al. 2023). When cells are exposed to external stimuli, L‐OPA1 is extensively degraded, preventing normal mitochondrial inner membrane fusion and resulting in mitochondrial fragmentation (Li, Xu, et al. 2022). This morphological abnormality is closely associated with neurodegenerative diseases (Chen, Shao, et al. 2023). Outer membrane fusion is a prerequisite for inner membrane fusion. MFN1 ensures the normal function of OPA1 by maintaining the mitochondrial membrane potential (Alghamdi 2024). Mitochondrial fission is mediated primarily by Drp1. The activity of Drp1 is regulated by both changes in its own spatial structure and posttranslational modifications (Kamerkar et al. 2025). When cells are at rest, Drp1 is distributed in the cytoplasm and lacks fission activity. Upon external stimulation, Drp1 undergoes dephosphorylation, is translocated to the OMM, and binds to receptors such as MFF and Fis1, thereby initiating mitochondrial fission (Xia et al. 2023). The various domains of Drp1 are responsible for regulating protein aggregation and mitochondrial membrane remodeling. Modifications such as AMPK‐mediated phosphorylation and SENP3‐mediated de‐SUMOylation alter Drp1 activity on the basis of cellular metabolic levels and stress states, ensuring that mitochondrial fission proceeds in an orderly manner (Das and Chakrabarti 2024). Upon cellular senescence, the levels of fusion‐associated proteins decrease, Drp1 activity abnormally increases, mitochondria exhibit fragmented morphologies, and mitochondrial physiological function gradually declines (Ajjan et al. 2026). This abnormality represents a systemic disruption of MFN, OPA1, and Drp1. Mutations in MFN2 and dysregulation of Drp1 both hinder the repair and clearance of damaged mitochondria, leading to the continuous accumulation of damage and subsequently triggering neurodegenerative diseases and cardiovascular age‐related pathologies (Camacho‐Encina et al. 2024) (Figure 3).

Additionally, mitochondrial fusion and fission work in concert with mitophagy to influence the aging process of cardiomyocytes. Mitochondrial fission induced by Drp1 can result in the isolation of dysfunctional mitochondria, thereby facilitating the normal activity of autophagy pathways such as PINK1/Parkin and BCL2‐interacting protein 3 (BNIP3)/BCL2‐interacting protein 3 like (NIX) (Ravindran and Gustafsson 2025). MFN2 can also bind to the microtubule‐associated protein 1 light chain 3 (LC3) protein to accelerate the degradation of damaged mitochondria (Ravindran and Gustafsson 2025). Cellular senescence disrupts these regulatory processes. Mitochondrial fission activity becomes excessively increased, while cellular autophagy activity continues to decline (Hong et al. 2025). In senescent cardiomyocytes, the expression levels of Parkin proteins decrease by 40%–60%, lysosomal acidification function becomes abnormal, and the expression levels of autophagy‐related 5 (Atg5) and autophagy‐related 7 (Atg7) proteins also change (Soh et al. 2023). Damaged mitochondria accumulate extensively within cells, releasing harmful substances that activate inflammatory responses and apoptosis pathways, ultimately leading to age‐related cardiovascular diseases such as cardiac hypertrophy, myocardial ischemia–reperfusion (I/R) injury, and heart failure (Chen et al. 2025). By integrating the interrelated roles of mitochondrial fusion and fission with autophagy, the molecular pathogenesis of age‐related cardiovascular diseases can be comprehensively elucidated.

## 4 Mitochondrial Dynamics Remodeling in Hypertension‐Induced Cardio‐Cerebral Comorbidity

The association between genetic variants in mitochondrial fusion genes and susceptibility to hypertension has been supported by multiple independent studies and exhibits significant age‐ and sex‐specific heterogeneity. A Korean study revealed significant correlations between *OPA1* gene single‐nucleotide polymorphisms (SNPs) and blood pressure levels as well as hypertension risk, with the association strength increasing with age, which was particularly pronounced in individuals older than 60 years (Jin et al. 2011). In the Chinese population, multiple Mfn2 gene SNP loci have been implicated in hypertension risk, with this association being particularly pronounced in men and exhibiting clear sex specificity. The frequency of risk alleles is significantly higher only in male patients, and specific haplotypes are more common in male patients. Moreover, another Chinese study demonstrated significantly elevated frequencies of multiple Mfn2 SNPs in male hypertensive patients, but these differences were not detected in females, and specific haplotypes were more common in male patients (Wang, Liu, et al. 2011). This sex difference may be influenced by the effect of MFN2 SNPs on lipid metabolism; metabolic disorders are associated with an increased risk of hypertension in men. OPA1 and Mfn2 genetic variants demonstrate differential associations across populations, ages, and sexes, with the strongest evidence observed in Chinese males (Li, Zhang, et al. 2016). For example, a novel 5′‐noncoding region variant at position −1248 A > G in Mfn2 was associated with hypertension risk in the Chinese population. Individuals carrying the G allele exhibited higher blood pressure, suggesting its potential involvement in pathogenesis through the regulation of gene expression. Variants in noncoding regions can reduce Mfn2 gene expression by disrupting transcriptional regulation, thereby weakening the inhibitory effect of this gene on the proliferation of vascular smooth muscle cells (VSMCs) and contributing to vascular remodeling.

A loss of function of mitochondrial fusion proteins disrupts fission–fusion homeostasis, leading to mitochondrial fragmentation and a burst of ROS, which have been identified as key drivers of vascular dysfunction in hypertension (Robert et al. 2021). At the molecular level, this imbalance is driven by a sophisticated yet vicious signaling network centered on the angiotensin II (AngII)–ROS–Drp1 axis (Ueda and Shibata 2024). Under hypertensive conditions, AngII increases the phosphorylation of serine at position 616 of dynamic protein‐related protein 1 (Drp1) in vascular, cardiac, and brain tissues via the angiotensin II type 1 receptor (AT1R)–protein kinase C (PKC)/extracellular signal‐regulated kinase (ERK) pathway while simultaneously inhibiting its phosphorylation at serine 637. This imbalance in phosphorylation sites constitutes the molecular switch that drives the translocation of Drp1 from the cytoplasm to the OMM and initiates fission (Preston et al. 2024). Concurrently, the substantial production of mitochondrial‐derived ROS synergistically amplifies damage through two pathways (Tian et al. 2026). On the one hand, it oxidatively modifies the Cys644 residue of Drp1, which directly increases its GTPase activity. On the other hand, it accelerates the K48‐ubiquitinated degradation of Mfn2 via the ubiquitin–proteasome pathway. Furthermore, ROS overexpress the mitochondrial inner membrane proteases OMA1 and YME1L, leading to abnormal cleavage of membrane‐bound long‐chain L‐OPA1 into soluble short‐chain S‐OPA1, thereby disrupting mitochondrial crista structure and inner membrane fusion function (Li, Li, et al. 2022; Richard et al. 2026). Under physiological conditions, mitochondrial fission and autophagy constitute an organelle homeostasis system in which small fragments generated by moderate fission can be recognized and cleared by the autophagy system (Castiglioni et al. 2024). However, in the pathological environment of hypertension, a decoupled state characterized by abnormally increased fission and reduced autophagic clearance capacity emerges; many fragmented mitochondria accumulate intracellularly and continuously release ROS, which further exacerbates fission disorders and results in the formation of a self‐reinforcing vicious cycle. This vicious cycle is prevalent in cardiac, vascular, and brain tissues and constitutes a common mechanism through which hypertension simultaneously induces multiorgan apoptosis and tissue dysfunction.

**FIGURE 4:**
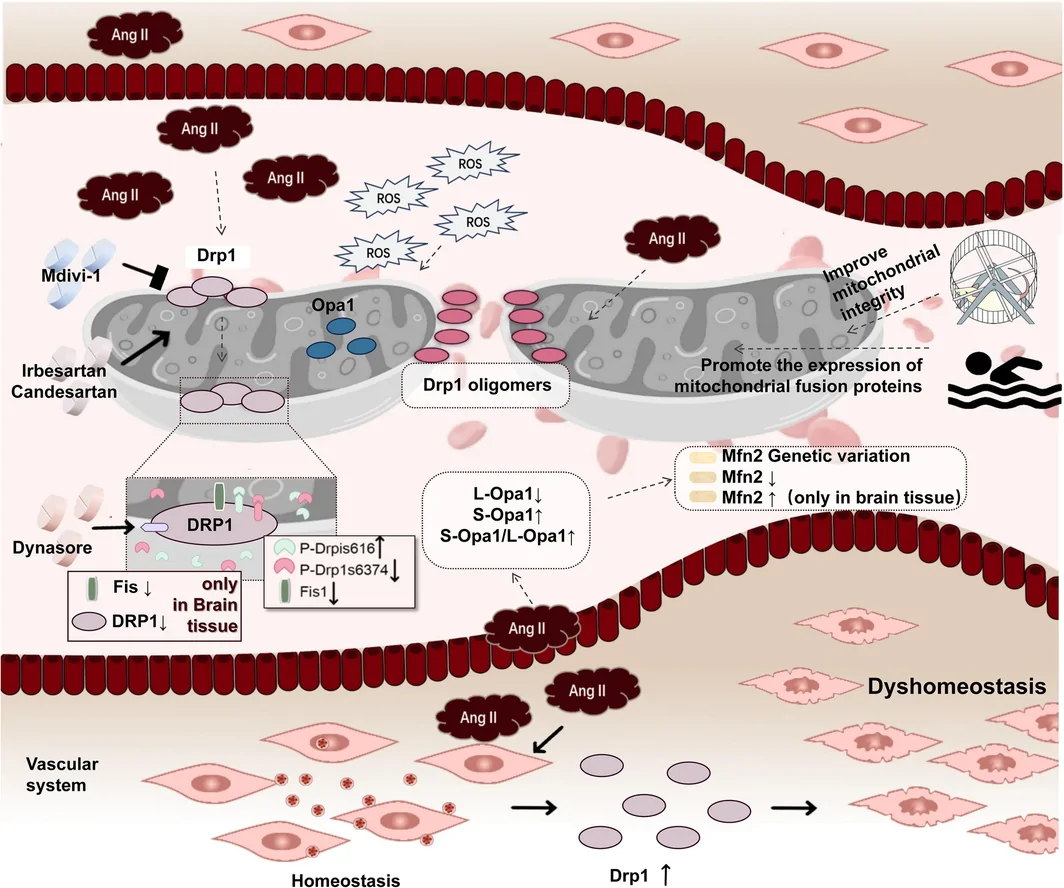
Multidimensional regulation of mitochondrial dynamics in hypertension. AngII promotes fission by increasing Drp1 Ser616 phosphorylation and decreasing Ser637 phosphorylation and inhibits fusion via Opa1 cleavage and ROS‐mediated Mfn2 degradation. Key proteins include fission mediator Drp1 and fusion factors Mfn1/2 (OMM) and Opa1 (IMM). Hypertension causes organ‐specific dysregulation: Drp1 is upregulated while Mfn2/Opa1 are downregulated in the heart and Mfn2 is upregulated while Drp1 is downregulated in the brain, leading to vascular remodeling, cardiomyocyte apoptosis, and microglial activation. Therapeutic approaches include Drp1 inhibitors, ARBs, and non‐pharmacological interventions, all aiming to restore mitochondrial dynamics balance.

Although the upstream damage pathways are similar, hypertension can induce distinct mitochondrial alterations in blood vessels, the heart, and the brain on the basis of the characteristics of the cells in each organ, their energy consumption patterns, and the local microenvironment. In smooth muscle cells and endothelial cells of resistance vessels, owing to their inherently low Mfn2 expression levels, these proteins are more susceptible to ROS‐induced ubiquitination and degradation. Furthermore, blood flow shear stress activates the translocation of Drp1 to the membrane; excessive fission ultimately promotes vasoconstriction, the proliferation of smooth muscle cells, and the disruption of the endothelial barrier (Abu‐Hanna et al. 2023; Preston et al. 2024; Robert et al. 2021). Mitochondria in the heart have almost completely lost the ability to fuse (Castiglioni et al. 2024). Cardiomyocytes are the cells with the highest mitochondrial density in the human body. Furthermore, the heart‐specific ubiquitin ligase Mul1 can simultaneously ubiquitinate and degrade Mfn1/2 and Opa1; the complete loss of fusion capacity ultimately induces irreversible myocardial hypertrophy and heart failure (Castiglioni et al. 2024). In contrast, brain tissue exhibits a phenotype characterized by insufficient fission and excessive fusion (Li, Li, et al. 2022). Hypertensive stimulation results in a significant increase in the expression of Mfn (fusion proteins), and brain‐derived neurotrophic factor (BDNF) specifically upregulates Mfn2 expression under hypertensive stress conditions to maintain long‐distance mitochondrial transport along axons (Figure 4).

Traditional small‐molecule drugs targeting Drp1 cannot distinguish between the physiological functions and pathological activities of Drp1 and often induce systemic side effects; therefore, drug development studies have shifted toward focusing on the regulation of Drp1 posttranslational modifications (Tian et al. 2026). Sirtuin 3 (SIRT3) catalyzes deacetylation at the K711 site of Drp1, thereby maintaining mitochondrial homeostasis. Under oxidative stress conditions, the acetylation levels at the K711 site of Drp1 abnormally increase, promoting Drp1 oligomerization and accelerating mitochondrial fragmentation. SIRT3 agonists remove the acetyl group, reducing abnormal Drp1 aggregation without interfering with its normal GTPase activity, thereby alleviating cytopathy caused by mitochondrial damage (Xi et al. 2025) and providing a structural reference for the development of conformation‐selective modulators. Mitochondrial–ER contact structures (MAMs) are also a potential research direction for cardiovascular disease interventions (Chen, Yang, Zhou, Yu, et al. 2024; Han et al. 2026). MAMs not only serve as carriers for anchoring Drp1 to the OMM but are also responsible for hypertension‐induced calcium overload and the substantial release of ROS. Hypertension‐induced metabolic disturbances disrupt the MFN2‐PACS2 complex and alter FUNDC1 expression via m6A methylation, leading to calcium overload; both types of damage result in abnormal Drp1 recruitment and excessive mitochondrial fission (Ma, Li, et al. 2025; Qu et al. 2025; Shen et al. 2024). Targeting the internal anchoring proteins of MAMs or modifying the associated catalytic enzymes can attenuate the pathological activation of Drp1 at the source of the signal, which can simultaneously improve intracellular calcium homeostasis and lipid metabolism. Compared with directly inhibiting Drp1 activity, this intervention strategy may be more applicable in cardiovascular remodeling. Related biophysics research can further enrich the design concepts for Drp. For example, the mechanism of Drp aggregation at fission sites can be explained in terms of liquid–liquid phase separation (Dridi et al. 2023; Piao et al. 2024). Additionally, the concentrations of intracellular ROS and calcium ions can alter the spatial conformation of the intrinsically disordered regions of Drp, leading to the formation of protein aggregates. At present, small‐molecule drugs capable of precisely regulating the properties of these aggregates are still in the early stages of development, and such formulations may result in excessive mitochondrial fission. By fine‐tuning the efficiency of mitochondrial fission by adjusting the physicochemical properties of these aggregates without completely inhibiting the overall enzymatic activity, the normal physiological functions of Drp could be preserved. This approach could reduce the risk of myocardial damage induced by long‐term medication use and drive the development of mitochondria‐targeted drugs toward biophysical regulation, moving beyond simple biochemical inhibition.

Genetic polymorphisms in mitochondrial fusion genes are major genetic risk factors for hypertension, and the dysregulation of mitochondrial fission and fusion mediated by the angiotensin II–ROS–Drp1 signaling axis is a core pathological mechanism underlying the multiorgan damage caused by hypertension. Given the limitations of traditional Drp‐targeted drugs, drug development focused on posttranslational protein modifications, organelle interactions, and biophysical regulation has become the main research direction in this field. The conclusions of existing genetic polymorphism studies are constrained by factors such as ethnicity and sex, limiting their generalizability; moreover, various candidate intervention strategies have been validated only in cell and animal models, and significant gaps remain before such strategies can be implemented in clinical practice.

## 5 Mitochondrial Dynamics in Age‐Related Cardiac Diseases

Disrupted mitochondrial dynamics mediated by Drp1 are correlated with the progression of age‐related cardiac diseases such as heart failure and myocardial infarction (Lu, Ma, et al. 2020). Furthermore, mitochondrial dysfunction is commonly associated with hypertension, and recent studies have increasingly indicated that disruption of mitochondrial dynamics plays a significant role in age‐related cardiac diseases. Changes in mitochondrial dynamics in age‐related cardiac diseases are summarized in Table 1.

**TABLE 1:** Mitochondrial dynamics in cardiovascular disease.

Disease	Model	Protein changes in disease	Mitochondrial morphological changes	References
Hypertension	SHRmitophagy+Ang II‐treated primary rat vascular smooth muscle cells (artery)	Drp1↑, Mfn2↓; Ang II‐induced Drp1 activation via ROS‐ERK1/2 pathway	Not described	(Zhang et al. 2018)
	SHR + Ang II‐treated human umbilical vein endothelial cells (artery)	Drp1↑, Opa1↓; Ang II promotes endothelial dysfunction via mitochondrial fission	Swollen/irregular cristae	(Li, Dang, et al. 2022)
	SHR (heart)	Mfn2↓, Opa1↓, s‐Opa1/l‐Opa1↑; imbalanced processing of Opa1	Not described	(Quiroga et al. 2020)
	SHR (heart)	Drp1↑, Mfn2↓, Opa1↓; AMPK activation rescues fusion/fission imbalance	Swollen, smaller mitochondria, disorganized cristae	(Hong et al. 2017)
	Ang II‐induced hypertensive mice (artery)	p‐Drp1 (Ser616)/Drp1↑; Phosphorylation of Drp1 at Ser616 is required for vascular smooth muscle cell (VSMC) proliferation	Not described	(Deng et al. 2021)
Atherosclerosis	HFD‐fed ApoE−/−mice+ox‐LDL‐treated RAW264.7 cells (vascular endothelium)	p‐Drp1 S616↑; Drp1 S616 phosphorylation promotes mitochondrial fission and endothelial damage	Morphological damage to mitochondria	(Su, Li, Wang, et al. 2023)
	HFD‐fed ApoE−/−mice (vascular endothelium)	Drp1↑, Fis1↑; hyperglycemia exacerbates fission via Drp1/Fis1	Small, punctate, short mitochondria	(Tong, Leng, et al. 2023)
Diabetic cardiomyopathy	STZ‐induced diabetic rats+H9C2 cells (heart)	Drp1↑; high glucose induces Drp1‐dependent fission	Smaller mean mitochondrial size	(Ding et al. 2018)
	STZ‐induced diabetic rats+primary cardiomyocytes (heart)	t‐Drp1↑, p‐Drp1 S616↑, p‐Drp1 S637↓, Mff↑, Mid51↑, Mid49↑; Mfn1↓, Mfn2↓, Opa1↓; comprehensive fission‐fusion imbalance	Smaller size, decreased number, cristae damage	(Feng et al. 2021)
	db/db mice+high glucose‐treated neonatal cardiomyocytes (heart)	Drp1↑, Fis1↑, L‐Opa1/S‐Opa1↓; Fis1 mediates fission via Opa1 cleavage	Fragmentation, increased number, decreased size	(Guo et al. 2020)
	db/db mice+HFD (heart)	Opa1↓; palmitate induces Opa1 loss leading to cristae disruption	Loss of electron density, ruptured/disorganized cristae	(Luo et al. 2024)
Myocardial infarction	LAD ligation in mice (heart)	p‐Drp1↑, Mff↑, Fis1↑; Mfn2↓, Opa1↓; multiple fission proteins activated	More mitochondrial fragmentation	(Liu et al. 2019)
	LAD ligation in rats (heart)	Drp1↓ (total) but GTP‐binding activity↑; activity‐dependent fission	Smaller, more rounded mitochondria	(Nishimura et al. 2018)
	LAD ligation in mice (heart)	Drp1↑, Fis1↑, Mff↑; Mfn1↓, Mfn2↓; coordinated upregulation of fission machinery	Morphological damage	(Wang, Zhuang, et al. 2024)
Myocardial ischemia–reperfusion	LAD ligation+I/R in mice (heart)	SUMO‐1/2/3 modified Drp1↑, Drp1/VDAC↑; SUMOylation promotes Drp1 translocation	Increased number, decreased size	(Shimizu et al. 2016)
	LAD ligation+I/R in rats (cardiac microvessels)	Mff↑; Mff overexpression exacerbates microvascular injury	Ruptured/vacuolated mitochondria	(Zhou et al. 2017)
	Langendorff I/R in rats (heart)	p‐Drp1/Drp1↑, cyto‐Drp1↓, mito‐Drp1↑; Opa1↓; Drp1 mitochondrial translocation	Edema, cristae disorganization	(Ciocci Pardo et al. 2019)
	H9C2 cells hypoxia‐reoxygenation (cardiomyocytes)	p‐Drp1 S616/Drp1↑, p‐Drp S637/Drp1↓; phosphorylation switch controls fission	Small, round, punctiform mitochondria	(Yang, Tian, et al. 2017)
Calcific aortic valve disease (CAVD)	Human VICs osteogenic differentiation (aortic valve)	p‐Drp1 S616/Drp1↑, p‐Drp1 S637/Drp1↓; phosphorylation switch controls fission	Sparse, fewer, empty mitochondria	(Liu et al. 2022)
Heart Failure	Failing human heart (ICM and DCM)	ICM: Drp1↑, Opa1↓, Mfn1↑, Mfn2↑; DCM: Drp1↑, Mfn1↑, Mfn2↑; differential fusion/fission profiles	Disorganized, smaller mitochondria, increased number	(Chen et al. 2009)
	LAD ligation‐induced HF in mice (heart)	p‐Drp1 S616/Drp1↑; Mfn2↓, L‐OPA1/S‐OPA1↓; persistent fission activation	Shortened length, increased vacuolar/swollen mitochondria	(Hu, Liu, et al. 2022)
	AAC‐induced HF in mice (heart)	Drp1↓, Fis1↓; Mfn1↓, Mfn2↓, Opa1↓; global downregulation of dynamics proteins	Poorly defined, disorganized networks	(Kim et al. 2020)
Post‐infarction remodeling	LAD ligation in SD rats (18 weeks) (heart)	Fis1↑, Mfn2↓, Opa1↓; sustained Fis1 upregulation and fusion loss	Not described	(Javadov et al. 2011)

### 5.1 Atherosclerosis (AS)

Multiple studies have consistently shown that mitochondrial dynamics are significantly disrupted in AS and that the expression or activation levels of pro‐fission proteins (Drp1 and Fis1) are increased, whereas the expression of pro‐fusion proteins (Mfn1, Mfn2, and Opa1) is decreased (Chen et al. 2017; Hasan et al. 2018; Preston et al. 2024; Su, Li, Shi, et al. 2023; Wang et al. 2017; Xue et al. 2019; Zhu, Liu, et al. 2019). This imbalance can be induced by factors such as apelin, oxidized low‐density lipoprotein (ox‐LDL), a high‐fat diet, and the AS‐promoting lipoprotein subtype L5 (Fang et al. 2022; Xie et al. 2020; Xue et al. 2019; You et al. 2023). Among these mechanisms, the primary pathways involve the miR‐93‐mediated silencing of Mfn2 (Feng et al. 2019) and the direct binding and activation of Drp1 by circHIPK3 (Li et al. 2025). Excessive activation of Drp1 not only directly mediates mitochondrial fragmentation but also promotes fragmentation by remodeling the ER structure and shortening the mitochondrial–ER junction (Montaigne et al. 2014). Conversely, the targeted inhibition of Drp1 reduces hepatic PCSK9 secretion and improves AS‐related lipid disorder‐induced damage. The complete mitochondrial quality control cycle also includes the selective clearance of fragmented mitochondria via mitophagy; dysfunction of this cycle further exacerbates the imbalance in mitochondrial dynamics, but the interaction between these two processes in AS remains poorly understood. In summary, an imbalance in mitochondrial fission/fusion is a central driver of abnormal proliferation of VSMCs in AS (Li, Xu, et al. 2022), and this phenomenon is highly consistent across clinical samples, animal models, and in vitro cellular experiments.

Recent in‐depth studies have revealed that increased mitochondrial fission in AS is a prerequisite for the “Warburg effect”‐like metabolic reprogramming of VSMCs (Sun et al. 2025). Under ox‐LDL or high glucose stimulation, the level of phosphorylation at the Drp1Ser616 site increases significantly, which not only mediates mitochondrial fragmentation but also directly promotes the transformation of VSMCs into synthetic, macrophage‐like, and osteogenic phenotypes (Mao et al. 2026; Meechem et al. 2022). For example, the membrane‐associated protein FAM177A1 induces mitochondrial oxidative damage and metabolic reprogramming by disrupting the SIRT3–SOD2 signaling axis, thereby driving the pathological transdifferentiation of VSMCs (Meechem et al. 2022). Furthermore, epigenetic modifications such as reduced DNA methylation caused by SAHH inhibition can specifically increase Drp1 expression in endothelial cells, resulting in the progression of metabolic stress to vascular aging and susceptibility to AS (You et al. 2023). Concurrently, defects in mitophagy lead to the accumulation of dysfunctional mitochondria, amplifying oxidative stress and metabolic dysregulation.

By targeting mitochondrial dynamics, anti‐atherosclerotic effects can be realized by regulating the phenotypes of immune and vascular cells in plaques; however, all existing intervention strategies are in the preclinical research stage. With respect to small‐molecule inhibitors, the Drp1 inhibitor Mdivi‐1 inhibits macrophage M1 polarization and promotes M2 reprogramming via the ROS/NLRP3 pathway, thereby alleviating intimal hyperplasia and plaque inflammation (Su, Li, Wang, et al. 2023). This approach has demonstrated promising therapeutic effects in multiple models, although its specificity remains to be validated. Additionally, melatonin inhibits macrophage mitochondrial fission and proinflammatory differentiation via the SIRT–Drp1 pathway, providing a theoretical basis for circadian rhythm‐targeted immunometabolic prevention and treatment of AS (Dai et al. 2026). With respect to traditional Chinese medicine and natural products, Buyang Huanwu Decoction inhibits excessive diabetes‐related mitochondrial fission and protects the endothelium via the AMPK–Drp1–Fis1 pathway (Tong, Leng, et al. 2023). Using liquid chromatography–mass spectrometry (LC–MS) and machine learning, nine active components, including methylniso‐lin, were identified, and these active compounds were linked to key mitochondrial targets (Zheng et al. 2025). *Centella asiatica* glycosides maintain mitochondrial homeostasis in VSMCs and inhibit abnormal cellular phenotypic transformation by blocking Drp1‐mediated mitochondrial translocation (Liu, Wang, et al. 2026). Owing to limitations in drug bioavailability and off‐target effects, targeted delivery and novel lead compounds have become key research focuses. The MITO Porter carrier enables the targeted delivery of coenzyme Q10 to mitochondria, increasing in vivo efficacy (Hibino et al. 2025). As a coenzyme Q10 derivative, idebenone inhibits the proliferation of smooth muscle cells via the PKM2‐mediated mitochondrial Hippo/YAP pathway while simultaneously improving statin‐induced mitochondrial damage (A. Xu et al., n.d.; Yu et al. 2024). Furthermore, the GOT2–AMPK–PPARα–CoQ pathway is involved in the regulation of ferroptosis and represents a potential target for stabilizing atherosclerotic plaques (H. Zhang et al. 2026). However, the in vivo safety, long‐term efficacy, and clinical translation potential of these novel strategies require further validation.

In summary, mitochondrial dysregulation is linked to metabolic remodeling and pathological phenotypic shifts across various cell types in the vascular wall. It serves as both the key pathological foundation for the onset and progression of AS and a common target for diverse drug classes, including small chemical molecules, natural plant monomers, traditional Chinese medicine formulations, and targeted nanomedicines. Existing intervention strategies have gradually expanded from simply inhibiting mitochondrial fission to diverse approaches, such as regulating immunometabolism and improving targeted mitochondrial delivery. However, owing to limitations in drug specificity, formulation stability, and clinical translation, further stratified and refined research is needed before these targeted therapies can be implemented in clinical practice.

### 5.2 Diabetic Cardiomyopathy

Diabetic patients exhibit impaired mitochondrial dynamics and myocardial contractile dysfunction, with mitochondria exhibiting an overall functional imbalance characterized by excessive fission and impaired fusion (Montaigne et al. 2014). In streptozotocin (STZ)‐induced animal models of type 1 and type 2 diabetes, the expression of the mitochondrial fusion‐related proteins Opa1 and Mfn1 is significantly reduced. Findings concerning Mfn2 expression vary, but its mRNA level has been shown to progressively decrease with disease progression, suggesting that Mfn2 may serve as a potential regulatory target at different stages of diabetic cardiomyopathy (Ding et al. 2020; Hagenbuchner et al. 2018; Yang, Yu, et al. 2017). These distinct results may be related to differences in experimental animal strains, the stage of diabetes progression, posttranslational ubiquitination and degradation of proteins, and variations in detection techniques. Metabolic reprogramming also participates in the regulatory process of mitochondrial fusion. The downregulation of PFKFB3 in the DCM state leads directly to the loss of Opa1 stability, further exacerbating mitochondrial fragmentation (Luo et al. 2024). In summary, the downregulation of Opa1 and Mfn1 has been reported consistently across diabetic myocardial fusion defects, whereas the activity of Mfn2 is influenced by multiple regulatory factors, resulting in inconsistencies between protein expression and gene transcription levels.

Among mitochondrial fission‐related proteins, the levels of Drp1 and its Ser616 phosphorylation (p‐Drp1S616) were significantly elevated, whereas Ser637 phosphorylation (p‐Drp1S637) was decreased (Feng et al. 2020). Furthermore, the secretion levels of other proteins, such as Fis1, Mff, Mid51, and Mid49, were significantly increased. Drp1 further exacerbates excessive mitochondrial fission while inducing ferroptosis in microvascular endothelial cells and thus serves as a key molecular switch responsible for microcirculatory dysfunction in DCM (Chen, Li, Guan, Yan, et al. 2024). The aforementioned protein expression and modification abnormalities have been validated in both STZ‐treated db/db mice and alloxan‐induced diabetic rat models (Hagenbuchner et al. 2018; Yang, Yu, et al. 2017). Furthermore, in db−/db− mouse myocardial microvascular endothelial cells, increased fatty acid β‐oxidation increases Opa1 and Mfn1 expression to promote mitochondrial fusion (Wang et al. 2023). Another study revealed that mitochondrial fission is activated in diabetic cardiomyopathy models and is accompanied by elevated levels of S‐nitrosylation of Drp1 (SNO‐Drp1), suggesting that SNO‐Drp1 may be a downstream target in diabetic cardiomyopathy with potential therapeutic value (Chen, Li, Guan, Yan, et al. 2024).

Interventions targeting mitochondrial dynamics have revealed that moderate‐intensity exercise reduces elevated Drp1 levels in diabetic cardiomyopathy models while maintaining the Mfn2/Drp1 ratio to mitigate disease progression (Veeranki et al. 2016). Melatonin prevents diabetes‐induced cardiac dysfunction by inhibiting Drp1‐mediated mitochondrial fission, and long‐term interventions delay disease progression by maintaining mitochondrial quality control (Ding et al. 2018; Yu et al. 2021). Multiple Chinese herbal components also show promise for treating diabetic cardiomyopathy through the regulation of mitochondrial dynamics. For instance, icariin alleviates diabetic cardiomyopathy symptoms by promoting mitochondrial fusion (Ni et al. 2020), whereas compounds such as paeoniflorin and pomegranate peel tannin exert cardioprotective effects by increasing Opa1‐mediated mitochondrial fusion (Fu et al. 2021; C. Liu et al. 2021). In this process, paeonol does not act directly on Opa1; instead, it acts as a specific ligand for CK2α to restore kinase activity, thereby activating the Janus kinase 2 (Jak2)/signal transducer and activator of transcription 3 (Stat3) signaling pathway. The activated Stat3 binds to the Opa1 promoter and increases its transcription, reversing the mitochondrial oxidative damage induced by high glucose levels at the molecular level (Liu et al. 2021). Additionally, ophiopogonin D improves myocardial function by inhibiting mitochondrial fission and promoting fusion (Zhang, Zhang, et al. 2022). Moreover, panax notoginseng total saponins act as dual‐action modulators that synergistically regulate metabolism and mitochondrial dynamics; they simultaneously rebalance the equilibrium between mitochondrial fission and fusion and correct myocardial lipid toxicity, thereby repairing both metabolic flux and mitochondrial morphology (Zhang, Zhang, et al. 2022). However, significant heterogeneity is observed among studies with respect to changes in Mfn2 expression, and conclusions are heavily influenced by factors such as animal strains, the disease stage, and detection methods. Furthermore, all investigations on the underlying mechanisms and effects of interventions have been limited to rodent models; evidence from clinical cohorts of patients with diabetic cardiomyopathy is lacking, and the optimal dosages and long‐term safety of various interventions have not been established.

### 5.3 Acute Myocardial Infarction

Acute myocardial ischemia induces significant mitochondrial fragmentation within cardiomyocytes, a process partially mediated by the transport of cyclin C from the nucleus to the cytoplasm, thereby promoting cell division (Ponce et al. 2020). Targeting this process to reduce mitochondrial fragmentation may serve as a therapeutic strategy to protect the heart from myocardial infarction injury (Ponce et al. 2020).

In animal models of myocardial infarction, mitochondria exhibit a typical dysbalanced phenotype characterized by excessive fission and impaired fusion. Studies have shown that the expression levels and Ser616 phosphorylation levels of Drp1 are elevated, the p‐Drp1S616/total Drp1 ratio is increased, and Drp1 is translocated from the cytoplasm to the mitochondria (Jiang et al. 2014; Lai et al. 2023; Liu et al. 2019; Qipshidze‐Kelm et al. 2014). The expression of Drp1 chaperones such as Fis1 and Mff is also increased, while the expression of the fusion proteins Mfn2 and Opa1 is decreased (Wang, Jiao, et al. 2011; Yang, Yu, et al. 2017), and the S‐Opa1/L‐Opa1 ratio is increased (Wang et al. 2019), indicating impaired inner membrane fusion function. However, some studies have reported reductions in total Drp1 protein levels but significantly increased GTP‐binding activity, which drives mitochondrial fission (Reshma et al. 2016), suggesting that posttranslational modifications and regulation of the activity of Drp1 may be the primary factors influencing pathological fission.

Intervention strategies for myocardial injury have increasingly involved multidirectional regulatory approaches. Mdivi‐1, a mitochondrial fission inhibitor developed to target Drp1, can inhibit abnormal mitochondrial fission in cardiomyocytes under hypoxic conditions (Reshma et al. 2016). However, these drugs exhibit significant off‐target toxicity, necessitating the development of safer, novel inhibitors. Drpitor1a, a highly specific Drp1 GTPase inhibitor, stabilizes mitochondrial calcium homeostasis during the prereperfusion phase of myocardial ischemia, prevents opening of the mitochondrial poration channel, and reduces the systemic toxicity associated with conventional drugs. Regulating the modification sites of the Drp1 protein is another viable approach; exogenous supplementation with disulfide donors can restore the redox state of the Drp1 Cys644 site. This intervention does not interfere with the protein's normal physiological functions and can block pathological mitochondrial fission (Piao et al. 2024). In studies targeting mitochondrial fusion and related receptors, ruscogenin has been shown to decrease Mfn2 levels within myocardial infarction lesions, which contributes to restoring normal mitochondrial fusion function (Liu, Zhao, et al. 2023). However, interventions focused on the expression of the Drp1 receptors MiD49 or MiD51 alone are insufficient to alleviate myocardial injury; the simultaneous silencing of both receptors is necessary to block the opening of the mitochondrial poration channel. Acute myocardial infarction leads to decreased MTFP1 expression; compared with increasing Mfn2 expression, using adeno‐associated virus 9 (AAV9) to increase the expression of this fusion cofactor yields better results with respect to the recovery of cardiac function (Hu et al. 2026; Samangouei et al. 2026). Additionally, conjugated linoleic acid possesses significant antioxidant capacity and can scavenge the large amounts of ROS that accumulate during myocardial injury, thereby maintaining the structural and functional stability of mitochondria and alleviating oxidative stress‐induced damage. Upon entering the body, sodium nitrite is converted into nitric oxide, which effectively inhibits pathological mitochondrial fission by inducing SNO‐Drp1. The combined use of these two compounds yields synergistic protective effects. By acting through two distinct pathways, namely, the regulation of oxidative stress and the remodeling of mitochondrial dynamics, they alleviate the damage caused by myocardial ischemia and reperfusion, thereby protecting cardiomyocytes and improving cardiac function. Dapagliflozin, a commonly used clinical antidiabetic drug, can also restore mitochondrial homeostasis, reduce myocardial apoptosis, and improve ventricular remodeling (Fan, Xu, et al. 2022). Drugs primarily exert their effects by holistically modulating mitochondrial dynamics, and related reports provide evidence for expanding their applications in the cardiovascular field.

In summary, although targeting mitochondrial dynamics is theoretically promising for realizing cardioprotective effects, significant gaps remain between current research and the clinical application of such strategies. Various interventions have demonstrated efficacy in preclinical models. However, most evidence was obtained from rodent or in vitro cellular models, and very few drugs targeting mitochondrial dynamics have actually progressed to clinical trials. Although the SGLT2 inhibitor dapagliflozin reduces the risk of hospitalization for heart failure and improves prognosis in clinical settings, its direct regulatory effect on mitochondrial dynamics is primarily inferred from mechanisms identified in basic research (Singh 2026). Furthermore, the changes in mitochondrial‐related miRNAs detected in patients with acute heart failure indicate only an association with pathological processes and cannot be used directly as diagnostic markers or therapeutic targets (Shirakabe et al. 2025). Therefore, future research should not be limited to mechanism validation; instead, translational studies guided by clinical hard endpoints should be conducted, and novel regulatory tools capable of being safely and specifically delivered to cardiac muscle mitochondria should be developed, thereby facilitating the translation of basic research findings into clinical applications (Qiu et al. 2025).

### 5.4 Myocardial I/R Injury

Myocardial I/R injury represents a common pathophysiological process in the diagnosis and treatment of cardiac disease. An imbalance in mitochondrial dynamics constitutes a key pathogenic mechanism. In myocardial I/R models, the expression and function of proteins associated with mitochondrial fission and fusion are significantly altered, thereby exacerbating myocardial cell damage and impairing cardiac functional recovery.

A consistent core phenotype of hyperproliferation has been observed across various animal and cellular I/R models, with significantly elevated phosphorylation levels of Drp1 and its Ser616 site, reduced phosphorylation levels at the Ser637 site, an imbalance in the ratio of p‐Drp1S616 to total Drp1, and a substantial translocation of Drp1 from the cytoplasm to the mitochondria (Chen, Chen, Wang, et al. 2020; Lee et al. 2020; Li, Yu, et al. 2020; Li et al. 2023; Ong et al. 2019; Palee et al. 2019; Piao et al. 2024; Shi et al. 2023; Wang, Jiao, et al. 2011). Furthermore, I/R injury is correlated with small ubiquitin‐like modifier (SUMO) modification of Drp1. In a mouse myocardial I/R model, the levels of SUMO‐1‐ and SUMO‐2/3‐modified Drp1 increased, the ratio of SUMO‐2/3‐modified Drp1 to SUMO‐1‐modified Drp1 increased, and mitochondrial Drp1 expression increased (Chen, Chen, Wang, et al. 2020; Li et al. 2023). The expression levels of Drp1‐interacting proteins such as Fis1 and Mff increase in a coordinated manner; among these proteins, phosphorylated Mff (p‐Mff) is key for the recruitment of cytosolic Drp1 to the mitochondria (Hall et al. 2021; Ji et al. 2022; Jin et al. 2018; Li et al. 2018; Li et al. 2023; Luo et al. 2024; Lu et al. 2018; Wang, Long, et al. 2012; Wu, Huang, et al. 2021). The mitochondrial metabolite succinate can trigger Drp1 translocation by promoting Mff phosphorylation, ultimately leading to mitochondrial dysfunction and cardiomyocyte apoptosis, suggesting a correlation between metabolic dysfunction and dynamic imbalance (Chen, Ma, Song, Hua, et al. 2024). Impaired fusion and excessive fission synergistically drive injury progression. The expression levels of Mfn1, Mfn2, and Opa1 are universally decreased in various I/R models, leading to mitochondrial fragmentation. However, Mfn2 plays a bidirectional role. Although Mfn1/Mfn2 double knockout in 4‐ to 6‐week‐old mice resulted in mitochondrial fragmentation and impaired baseline cardiac function, the infarct size was significantly smaller than that in wild‐type mice. Moderate downregulation of Mfn2 actually protected cardiomyocytes from lethal hypoxia–reoxygenation injury, suggesting that the regulation of fusion proteins must balance baseline physiological function with pathological stress states (Hall et al. 2021).

Based on the aforementioned mechanisms, targeting mitochondrial fission and fusion has emerged as a key therapeutic strategy for myocardial I/R injury. Drp1, a core protein that mediates mitochondrial fission, can be inhibited by the small‐molecule inhibitor Mdivi‐1, thereby blocking its translocation to mitochondria and mitigating I/R‐induced mitochondrial fragmentation and apoptosis. Loading Mdivi‐1 into nanoparticle delivery systems further increases inhibitory effects, as demonstrated by the ability of MK‐886 to protect mouse cardiac function through the downregulation of abnormally elevated Drp1 expression (Shi et al. 2023). However, the clinical translation of these broad‐spectrum inhibitors has been limited by their off‐target effects and the risk of long‐term inhibition‐induced cardiomyopathy. Drpitor1a, a new‐generation, highly selective GTPase inhibitor, can specifically block Drp1‐mediated mitochondrial calcium overload and ROS bursts prior to reperfusion without affecting baseline cardiac contractile function and may thus represent an effective solution to the safety challenges associated with traditional inhibitors (Piao 2023; Piao et al. 2024). The differential regulation of phosphorylation sites represents another new direction. The AMP‐activated protein kinase (AMPK) activator AICAR corrects kinetic dysregulation in a bidirectional manner by inhibiting phosphorylation at the mitogenic site Ser616 and increasing phosphorylation at the antimitogenic site Ser637 while simultaneously improving mitophagy flux and the inflammatory microenvironment (Palee et al. 2019; Shi et al. 2023). The MCU inhibitor Ru360 also increases Opa1 levels and blocks Drp1 translocation, further improving cardiac function (García‐Rivas et al. 2006). Additionally, metabolic modulators such as PCSK9 inhibitors (PCSK9i) and the proteasome inhibitor MG132 can reverse the cardiac dysfunction caused by reduced fusion. Melatonin exerts cardioprotective effects by activating OPA1 and balancing mitochondrial dynamics, whereas vitamin D3 and choline have beneficial effects on mitochondrial function and provide additional protective benefits (Dal Zotto et al. 2021; Ma and Dong 2019; Sonobe et al. 2025). Lifestyle interventions also exert effects. For example, aerobic exercise reduces Drp1 mRNA expression and mitigates I/R injury (Luo et al. 2021), whereas therapeutic hypothermia (TH) inhibits mitochondrial translocation by blocking dephosphorylation at the Drp1 S637 site (Sharp et al. 2014). Furthermore, multiple active components from traditional Chinese medicine, such as baicalin, astragaloside IV derivatives, omegastrol, and ursolic acid, have been demonstrated to correct I/R‐induced mitochondrial dysfunction and exert cardioprotective effects (Chen, Chen, Wang, et al. 2020; Khuanjing et al. 2021; Lahnwong et al. 2020; Li, Yu, et al. 2020; Liu, Wei, et al. 2024; Yu et al. 2022). The compound GRS and Shengmai preparations inhibit Drp1 translocation in cardiomyocytes under hypoxic–reoxygenation conditions (Xiong et al. 2023). In summary, inhibiting Drp1‐mediated mitochondrial fission or promoting Opa1/Mfn‐mediated fusion, combined with metabolic regulation and lifestyle interventions, constitutes the primary therapeutic strategy for ischemic–reperfusion‐induced myocardial injury.

In addition to these interventions targeting core mechanisms associated with mitochondrial dynamics, drugs from diverse therapeutic fields have demonstrated cross‐disciplinary cardioprotective effects. In neurology, donepezil, a drug for treating AD, has been shown to restore the balance of mitochondrial dynamics in myocardial I/R model rats, reducing the infarct size, suppressing reperfusion arrhythmias, and improving left ventricular systolic and diastolic function (Zou et al. 2022). Among antidiabetic drugs, metformin directly improves mitochondrial function in I/R model rats, thereby protecting cardiac function (Cai et al. 2022). Dapagliflozin, through pretreatment, increases Opa1 expression, enhances mitochondrial function, and reduces myocardial apoptosis, thereby providing potent protective effects against myocardial I/R injury and ultimately improving left ventricular function (Kalkhoran et al. 2022). Moreover, empagliflozin specifically protects myocardial microvascular endothelial cells during I/R injury, maintaining microcirculatory stability (Liu et al. 2022). Among antihypertensive drugs, hydralazine not only inhibits oxidative stress‐induced mitochondrial fission and membrane depolarization in HeLa cells (Kalkhoran et al. 2022) but also significantly reduces the myocardial infarction area in both in vitro and in vivo models of acute I/R injury through pretreatment mechanisms that decrease mitochondrial fission and preserve mitochondrial membrane integrity. These findings demonstrate the potential therapeutic value of cross‐disciplinary drugs in treating myocardial I/R injury.

However, most preclinical intervention strategies that have shown efficacy have not been fully replicated in human trials. Animal models lack comorbidities such as hypertension and diabetes; modeling methods differ from clinical procedures; and existing studies generally lack assessments of hard endpoints such as long‐term heart failure and mortality. In terms of drug development, the first‐generation Drp1 inhibitor Mdivi‐1 is rarely used in clinical practice because of its off‐target effects and potential cardiotoxicity. Although newer, highly selective inhibitors such as Drpitor1a offer improved safety, they remain in the early preclinical stages and have not yet entered phase I human trials (Liu, Huang, et al. 2026; Wu et al. 2020). Although metformin and SGLT2 inhibitors have been shown to reduce the risk of MACE in large‐scale CVOTs, the mechanism through which they exert specific I/R protective effects through mitochondrial dynamics has been validated only in animals, and large‐scale RCT evidence regarding reperfusion injury in acute myocardial infarction is lacking (H. Kim et al. 2025). Moreover, limited clinical data is available for melatonin; the benefits of melatonin in CABG patients are limited to improvements in perioperative biomarkers, and no studies have reported a clear regulatory mechanism (Casper et al. 2025; Ibrahim et al. 2022).

### 5.5 Heart Valve Disease

The calcification of heart valves induces valvular interstitial cells (VICs) to differentiate into myofibroblasts, leading to diffuse calcification or osteogenic differentiation. We isolated primary human valve interstitial cells from aortic valve leaflets and induced osteogenic differentiation of vascular endothelial cells using β‐glycerophosphate sodium (b‐GA), dexamethasone, and ascorbic acid (b‐GA system) while inducing myofibroblast differentiation using recombinant human transforming growth factor‐β (TGF‐β). In this model, the expression of Drp1 and L‐Opa1 was increased, whereas S‐Opa1 levels did not significantly change. These findings suggest that mitochondrial dynamics‐related proteins may be involved in maintaining the proliferation of VICs and aortic valve homeostasis in aortic valve disease.

The protein tyrosine phosphatase 1B (PTP1B)‐specific inhibitor MSI‐1436 modulates the L‐Opa1/S‐Opa1 ratio in mitochondrial dynamics, thereby affecting the osteogenic differentiation of VICs (Chang et al. 2022). If such modifications are targeted at only specific sites on Drp1, more precise effects can be achieved. In vitro models have demonstrated that peroxynitrite disrupts mitochondrial dynamics by nitrosylating Drp1 at tyrosine 628 (Y628), thereby promoting the osteogenic differentiation of VICs (Huang et al. 2021). Furthermore, deacetylases such as SIRT1 (Xu et al. 2024), SIRT3 (Kaur et al. 2024), and SIRT5 (Ke et al. 2025) can reversibly restore the physiological function of Drp1 through posttranslational modifications in cardiac mitochondrial dynamics, potentially yielding better results than complete knockout or inhibitor‐mediated approaches. This strategy integrates various pathological signals involved in disease progression, providing potential targets for the development of allosteric modulators of Drp1 and site‐specific peptide therapeutics. A high‐fructose diet can be used to establish a model of metabolic syndrome and mimic the pathological features of aortic stenosis. In this model, the expression of DRP1 and Opa1 is significantly elevated; these two proteins can prematurely activate the PI3K/mitochondrial signaling pathway, inducing osteogenic differentiation in human valvular mesenchymal cells (Wei et al. 2021). Calcific aortic valve disease (CAVD) is a classic example of a metabolism‐related disorder in which metabolic stress is the primary cause of mitochondrial dysfunction. Therefore, the screening of upstream targets in the pathological cascade should be prioritized in drug development. Targeting the gene *MGST1*, which encodes the mitochondrial–ER contact site (Mai et al. 2026), or regulating the mechanometabolic signaling interaction mediated by the Piezo1 protein can effectively inhibit the progression of valvular calcification (Zhong et al. 2023).

Current research has elucidated the theoretical role of mitochondrial dysregulation in osteogenic differentiation and metabolic–mechanical coupling mechanisms in CAVD. However, significant challenges in clinical translation must be addressed. Although single‐cell transcriptomic analysis has confirmed the differential expression of the mitochondrial‐associated genes MGST1 and Piezo1 in human calcified valve tissue, most intervention targets have not yet been supported by reliable clinical evidence in humans. Specifically, interventions targeting the Drp1 Y628 nitroylation site have been validated only in vitro and in rodent models. Additionally, evidence from randomized controlled trials on the efficacy of tetrahydrobiopterin is lacking, and the regulatory role of the SIRT family on Drp1 has not been confirmed in large‐scale clinical cohorts. Furthermore, although the high‐fructose‐activated PI3K/mitochondrial pathway has been confirmed in human cells, single‐nutrient models cannot fully reflect the characteristics of the multiple metabolic dysregulations involved in CAVD. Factors such as sex may also significantly influence the efficacy of mitochondria‐targeted therapies. Overall, this field is currently at a critical juncture, transitioning from mechanistic exploration to clinical validation. Translational studies using human valve tissue should be prioritized to accurately assess the clinical significance of key targets and bridge the gap between basic research and clinical evidence.

### 5.6 Other Considerations

The characteristics of cardiac aging include myocardial hypertrophy, fibrosis, the accumulation of misfolded proteins, and mitochondrial dysfunction. Pathological myocardial hypertrophy associated with aging is considered an inevitable precursor to heart failure (Javadov et al. 2011). Reduced Drp1 expression and decreased p‐Drp1S616 levels in aged hearts lead to increased mitochondrial damage and apoptosis (Shou and Huo 2022). Following myocardial infarction, mouse hearts undergo remodeling characterized by impaired cardiac contractility, enlarged infarct sizes, and significant myocardial interstitial fibrosis, accompanied by increased apoptosis and mitochondrial damage (Hu, Zhang, et al. 2020). In Sprague–Dawley (SD) rats undergoing coronary artery ligation‐induced ventricular remodeling, Fis1 expression increased at 12 weeks, whereas Mfn2 expression decreased; by 18 weeks, Fis1 expression remained elevated, whereas Mfn2 and Opa1 expression were reduced (Hu, Zhang, et al. 2020). This study revealed that postinfarction remodeling alters the expression of proteins associated with mitochondrial fission and fusion, with particularly pronounced changes in the expression of Fis1 and Mfn2. Melatonin alleviates myocardial infarction injury and postinfarction remodeling by regulating Mfn2 expression.

In hypertension‐induced heart failure with a reduced ejection fraction (HFrEF), p‐Drp1S616 levels decrease; this reduction impairs Drp1 mitochondrial localization, inhibits mitochondrial fission, and ultimately leads to mitochondrial dysfunction (El‐Sayed et al. 2024). Lipid overload promotes Drp1 acetylation, which subsequently causes cardiac dysfunction (Kim et al. 2020). In an isoproterenol hydrochloride‐induced rat heart failure model, Drp1 expression was increased, whereas Mfn2 expression was decreased (Chen et al. 2009). Furthermore, in aortic coarctation (AAC)‐induced heart failure mice, the expression of the mitochondrial fusion‐associated protein Mfn2 was significantly downregulated, and MitoQ (a mitochondrial‐targeted antioxidant) alleviated Mfn2 downregulation and exerted cardioprotective effects (Ferreira et al. 2019).

Additionally, in a rat model of ischemic cardiomyopathy induced by coronary artery ligation, Mfn1 and Mfn2 expression increased, whereas Opa1 expression decreased (Hu, Liu, et al. 2022). The phosphorylation of Mfn1 leads to the partial loss of its GTPase activity, which results in mitochondrial fragmentation and dysfunction, which may explain the impaired mitochondrial fusion observed during heart failure (Chaanine et al. 2019). Moreover, in a coronary artery ligation‐induced ischemic heart failure mouse model, Mfn2 expression decreased, the L‐Opa1/S‐Opa1 ratio decreased, and the p‐Drp1S616/Drp1 ratio increased (Campos et al. 2017). Variations in mitochondrial dynamics have been observed across different animal models, and these differences are also observed in human heart failure caused by various diseases. For example, in heart failure induced by ischemic cardiomyopathy, Opa1 expression is decreased, whereas Mfn1, Mfn2, and Drp1 expression is increased; conversely, in heart failure induced by nonischemic cardiomyopathy, Mfn1 and Mfn2 expression is increased, whereas Drp1 expression does not significantly change (de la Cueva et al. 2022). Consequently, Opa1 expression is reduced in both animal and human models of ischemia‐induced heart failure, and inhibiting Opa1 expression exacerbates mitochondrial fragmentation and increases apoptosis (Hu, Liu, et al. 2022). Furthermore, in a previous study, researchers collected subepicardial left ventricular biopsy samples from patients with heart failure with reduced ejection fraction (HFrEF). Among these patients, 4 underwent aortic valve replacement (AVR), 5 underwent coronary artery bypass grafting (CABG), and 4 underwent left ventricular assist device (LVAD) implantation (Ribeiro et al. 2019). Drp1 levels were significantly elevated in the AVR and CABG groups but not in the LVAD group (de la Cueva et al. 2022).

Melatonin and captopril (used alone or in combination) exert beneficial effects on mitochondrial dynamics in hypertension‐induced heart failure models, providing cardioprotective effects (L. Chen et al. 2009). Furthermore, physical exercise significantly and synergistically improves mitochondrial quality control and bioenergetic efficiency in patients with ischemic heart failure, thereby enhancing clinical outcomes (Ferreira et al. 2019).

Treatment strategies for age‐related cardiac diseases are summarized in Table 2.

**TABLE 2:** Treatment of aging‐related heart diseases.

Disease	Description	Intervention	Model	Effects on mitochondrial dynamics	Protein changes	References
Hypertension	Selective inhibitor of Drp1	mdivi‐1	Inhibition of ET‐1 induced mesenteric artery vasoconstriction in rats	Inhibits fission	Drp1 ↓	(Basu et al. 2017)
			Ang II‐induced hypertension and cardiovascular remodeling	Inhibits fission	Drp1 ↓	(Montaigne et al. 2014)
			Spontaneously hypertensive Rats; Angiotensin II‐treated primary rat vascular smooth muscle cells	Inhibits fission	Drp1 ↓	(Wang, Long, et al. 2012)
			Hypertensive cardiac hypertrophy and fibrosis in Dahl‐salt sensitive rats	Inhibits fission	Drp1 ↓	(Ding et al. 2021)
			Hypertensive human; Spontaneously hypertensive Rats; Angiotensin II‐induced hypertensive Mice	Inhibits fission	Drp1 ↓	(Li et al. 2021)
			Spontaneously hypertensive Rats; Angiotensin II–treated primary neonatal rat cardiomyocytes	Inhibits fission	Drp1 ↓	(Chen, Li, et al. 2022)
			Angiotensin II‐induced hypertensive mice; Angiotensin II–treated primary vascular smooth muscle cells	Inhibits fission	Drp1 ↓	(Lu, Qi, et al. 2020)
	Anti‐hypertensive drug	Irbesartan	Spontaneously hypertensive Rats; Angiotensin II–treated primary neonatal rat cardiomyocytes	Inhibits fission	Drp1 ↓	(Chen, Li, et al. 2022)
		Candesartan	Spontaneously hypertensive Rats	Promotes fusion	Mfn2↑	(Wang, Zhuang, et al. 2024)
	Exercise	Swimming	Hypertensive human; Spontaneously hypertensive Rats; Angiotensin II‐induced hypertensive Mice	Inhibits fission	Drp1 ↓	(Li et al. 2021)
		Voluntary wheel running exercise training	Angiotensin II‐induced hypertensive mice; Laminar shear stress (20 dyne/cm2) was applied to Human aortic endothelial cells (HAECs)	Promotes fusion	Mfn1↑, Mfn2↑	(Liu, Zhao, et al. 2023)
	Chinese medicine prescriptions and active extracts	Pomegranate extract	Spontaneously hypertensive Rats	Inhibits fusion	Mfn2↓	(Yang, Tian, et al. 2017)
	Others	FK506 and CSA	Angiotensin II‐induced hypertensive mice; Angiotensin II–treated primary adventitial fibroblasts	Inhibits fission	Inhibition of p‐Drp1^S637^	(Chen, Chen, Chan, et al. 2020)
		Dynasore	ET‐1 induced mesenteric artery vasoconstriction in rats	Inhibits fission	Drp1 ↓	(Basu et al. 2017)
		L‐2286	Spontaneously hypertensive Rats; H_2_O_2_‐treated neonatal rat cardiomyocytes	Inhibits fission	Inhibit Drp1 translocation; Mfn2↑, Opa1↑	(Franco et al. 2023)
Atherosclerosis	Selective inhibitor of Drp1	Mdivi‐1	High fat diet (HFD) fed APOE−/− mice; ox‐LDL‐treated RAW264.7 cells	Inhibits fission	p‐Drp1^S616^↓	(Reshma et al. 2016)
	Chinese medicine prescriptions and active extracts	Buyang huanwu decoction	High fat diet (HFD) fed APOE−/− mice treated with STZ	Inhibits fission	Drp1↓, Fis1↓	(Wang, Jiao, et al. 2011)
	Others	CoQ10	High fat diet (HFD) fed APOE−/− mice; ox‐LDL‐treated Human aortic endothelial cells	Promotes fusion	Opa1↑	(Chen, Chen, Wang, et al. 2020)
Diabetic cardiomyopathy	Selective inhibitor of Drp1	mdivi‐1	Leptin receptor‐deficient (db−/db−) mice; neonatal rat cardiomyocytes treated with glucose	Inhibits fission	p‐DRP1^S616^↓; p‐DRP1^S637^↑	(Ciocci Pardo et al. 2019)
	Mitochondrial fusion promoter	M1	STZ‐induced diabetes rats; primary cardiomyocytes treated with different concentrations of glucose	Promotes fusion	Opa1↑	(Huang et al. 2019)
	Chinese medicine prescriptions and active extracts	Icariin	Leptin receptor‐deficient (db−/db−) mice; primary cardiomyocytes treated with different concentrations of glucose	Promotes fusion	Mfn2↑	(Tong, Mukai, et al. 2023)
		Paeonol	STZ‐induced diabetes rats; primary neonatal cardiomyocytes treated with glucose	Promotes fusion	Opa1↑	(Luo et al. 2024)
		Ophiopogonin D	Leptin receptor‐deficient (db−/db−) mice	Inhibits fission, promotes fusion	p‐Drp1^S616^/Drp1↓; Mfn1↑, Mfn2↑, Opa1↑	(Ordog et al. 2021)
		Punicalagin	STZ‐induced diabetes rats; primary neonatal cardiomyocytes treated with glucose	Promotes fusion	Opa1↑	(Zhu, Liu, et al. 2019)
		Fufang Zhenzhu Tiaozhi	STZ‐induced diabetes mice	Inhibits fission, promotes fusion	Drp1↓, Fis1↓; Mfn2↑, Opa1↑	(Ong et al. 2019)
	Others	RTA 408	STZ‐induced diabetes mice; Leptin receptor‐deficient (db−/db−) mice; primary neonatal mouse cardiomyocytes and H9C2 treated with glucose	Inhibits fission, promotes fusion	Drp1↓; Mfn1↑, Mfn2↑, Opa1↑	(Olmedo et al. 2020)
		Melatonin	STZ‐induced diabetes mice; H9C2 cells treated with different concentrations of glucose	Inhibits fission	Drp1↓	(Deng et al. 2021)
Myocardial infarction	Selective inhibitor of Drp1	Mdivi‐1	H9C2 cells treated with oxygen–glucose deprivation	Inhibits fission	p‐Drp1^S616^/Drp1↓, p‐Drp1^S637^/Drp1↑	(Du et al. 2022)
	Hypoglycemic drug	Dapagliflozin	The left anterior descending (LAD)coronary artery was ligation in SD rats	Normalizes the mitochondrial fission	Drp1↑; Mfn2↑	(Wang et al. 2016)
	Exercise	Aerobic Interval Training (AIT)	The left anterior descending (LAD)coronary artery was ligation in Rats	Promotes fusion, inhibits fission	Drp1↓; Mfn2↑, Opa1↑	(Ding et al. 2020)
	Chinese medicine prescriptions and active extracts	*Tribulus terrestris* L. fruit methanol extract	H9C2 cells treated with hypoxia	Promotes fusion, inhibits fission	Drp1↓, Fis1↓; MFN2↑, OPA1↑	(Zhang, Zhang, et al. 2024)
		Extract of Sheng‐Mai‐San (ESMS)	H9C2 cells treated with oxygen–glucose deprivation	Inhibits fission	p‐Drp1^S616^/Drp1↓, p‐Drp1^S637^/Drp1↑	(Du et al. 2022)
		Ruscogenin	The left anterior descending (LAD)coronary artery was ligation in ICR mice	Promotes fusion, inhibits fission	p‐Drp1^S616^/Drp1↓; Mfn2↑, S‐OPA1/L‐OPA1↓	(Wang et al. 2023)
	Others	Conjugated linoleic acid (cLA) and nitrite	The left anterior descending (LAD)coronary artery was ligation in C57BL/6 mice	Inhibits fission	Drp1↓	(Rohani et al. 2020)
		7,8‐dihydroxyflavone (7,8‐DHF)	The left anterior descending (LAD)coronary artery was ligation in Kunming mice	Inhibits fission	Fis1↓	(Shi et al. 2023)
Myocardial ischemia–reperfusion	Selective inhibitor of Drp1	Mdivi‐1	Langendorff‐Perfused Wisrar rats Heart IR Model	Blocked dephosphorylation of Drp1 S637 and mitochondrial translocation of DRP1	p‐Drp1^S637^/Drp1↑, p‐Drp1^S637^↑, Mito‐Drp1↓	(Jiang et al. 2014)
			HL‐1 cells treated with hypoxia reoxygenation	Inhibits the translocation of Drp1 to mitochondria	Mito‐Drp1↓	(Nandi et al. 2021)
			The left anterior descending (LAD)coronary artery was ligation in C57BL/6 mice	Inhibits the translocation of Drp1	Mito‐Drp1↓	(Zhang, Feng, et al. 2022)
		Mdivi‐1; PLGA‐NP‐mediated delivery of Mdivi1 (Mdivi1‐NP)	Langendorff‐Perfused Mouse Heart IR Model	Inhibits the translocation of Drp1 to mitochondria	Mito‐Drp1↓	(Rogers et al. 2021)
	Mitochondrial fusion promoter	M1	The left anterior descending (LAD)coronary artery was ligation in Wistar Rats	Promotes fusion	Mito:Mfn2↑, Opa1↑	(Chen et al. 2011)
	Mitochondrial calcium uniporter inhibitor	Ru360	The left anterior descending (LAD)coronary artery was ligation in C57BL/6 mice; neonatal rat ventricular cardiomyocytes treated with hypoxia reoxygenation	Inhibits the translocation of Drp1, Promotes fusion	Cyto‐Drp1↑, Mito‐Drp1↓, Mito‐Opa1↑	(Tong, Leng, et al. 2023)
	Lipid‐lowering drug	PCSK9 inhibitor (PCSK9i)	The left anterior descending (LAD)coronary artery was ligation in Wistar Rats	Promotes fusion, inhibits fission	Mito:p‐Drp1^S616^/Drp1↓; Mfn2↑	(Kim et al. 2015)
	Hypoglycemic drugs	Metformin	The left anterior descending (LAD)coronary artery was ligation in Wistar Rats	Inhibits fission	Mito‐Drp1↓	(Papanicolaou et al. 2011)
		Empagliflozin	The left anterior descending (LAD)coronary artery was ligation in C57BL/6 mice	Promotes fusion, inhibits fission	p‐Drp1/Drp1↓, Fis1↓; Mfn2↑, Opa1↑	(Lee et al. 2016)
		Empagliflozin	Human coronary artery endothelial cells treated with H_2_O_2_	Inhibits the translocation of Drp1	p‐Drp1/Drp1↓, p‐Fis1/Fis1↓, mito‐Drp1↓, cyto‐Drp1↑	(Zhou, Wu, et al. 2019)
	Anti‐dementia drug	Donepezil	The left anterior descending (LAD)coronary artery was ligation in Wistar Rats	Inhibits the translocation of Drp1; Promotes fusion	Cyto:p‐Drp1^S616^/Drp1↓, Mito:Mfn2↑, Opa1↑	(Feng et al. 2021)
	Exercise	Aerobic exercise	The left anterior descending (LAD)coronary artery was ligation in C57BL/6 mice	Inhibits fission	Drp1 mRNA↓; Mfn2 mRNA↑	(Ren et al. 2023)
		Aerobic exercise	The left anterior descending (LAD)coronary artery was ligation in C57BL/6 mice	Promotes fusion, inhibits fission	Drp1↓; Mfn2↑	(Gallo et al. 2024)
	Chinese medicine prescriptions and active extracts	GRS: the proportion as 6:0.75:6 including ginsenoside Rb1, ruscogenin, and schisandrin	Neonatal rat ventricular myocytes treated with hypoxia reoxygenation	Inhibits the translocation of Drp1	p‐Drp1/Drp1↓	(Guo et al. 2020)
		Shengmai preparations (YiQiFuMai powder injection)	Neonatal rat ventricular myocytes treated with hypoxia reoxygenation	Inhibits the translocation of Drp1	p‐Drp1/Drp1↓	(Guo et al. 2020)
		Vitexin	Langendorff‐Perfused SD rats Heart IR Model H9C2 cells treated with hypoxia reoxygenation	Promotes fusion, inhibits fission	mito‐Drp1↓; Mfn2↑	(Lee et al. 2020)
	
		LS‐102 Astragaloside IV derivative	The left anterior descending (LAD)coronary artery was ligation in SD rats: H9C2 cells treated with hypoxia reoxygenation	Inhibits fission	p‐Drp1^S616^↓, p‐Drp1^S637^↑	(Jiang et al. 2015)
		Baicalein	The left anterior descending (LAD)coronary artery was ligation in C57BL/6 mice	Inhibits fission	Drp1↓	(Zhou et al. 2024)
		*Lycium barbarum* polysaccharide (LBP)	The left anterior descending (LAD)coronary artery was ligation in SD rats	Promotes fusion, inhibits fission	Drp1↓; Mfn2↑, Opa1↑	(Ni et al. 2020)
		Ursolic Acid	The left anterior descending (LAD)coronary artery was ligation in C57BL/6J mice	Promotes fusion, inhibits fission	Drp1↓; Mfn1↑, Mfn2↑	(Li, Yu, et al. 2020)
		Longxuetongluo Capsule (LTC)	The left anterior descending (LAD)coronary artery was ligation in Wistar rats	Promotes fusion, inhibits fission	p‐Drp1/Drp1↓, Mfn2↑	(Xue et al. 2020)
		Shuangshen Ningxin Formula (SSNX)	The left anterior descending (LAD)coronary artery was ligation in SD rats	Inhibits fission	Drp1↓, Mff↓	(Chen, Li, Guan, Yan, et al. 2024)
	Isoliquiritigenin (ISL)	The left anterior descending (LAD)coronary artery was ligation in C57BL/6 mice	Inhibits fission	Drp1↓	(Dong et al. 2016)
	Others	MK‐886 (an inducer of cardiac proteasome expression and activity)	The left anterior descending (LAD)coronary artery was ligation in C57BL/6J mice	Promotes fusion, inhibits fission	Drp1↓, p‐Drp1^S616^↓; Mfn1↑, Mfn2↑	(Lai et al. 2023)
		Blebbistatin (myosin II inhibitor)	H9C2 cells treated with hypoxia reoxygenation	Inhibits fission	p‐Drp1/Drp1↓	(Wu, Zheng, et al. 2021)
		AICAR (the AMPK activator)	Langendorff‐Perfused mice Heart IR Model; H9C2 cells treated with hypoxia reoxygenation	Promotes fusion, inhibits fission	In vivo:p‐Drp1^S616^/Drp1↓, Mfn1 mRNA↑, in vitro:p‐Drp1^S616^/Drp1↓, p‐Drp1^S637^/Drp1↑, Fis1 mRNA↓, Mff mRNA↓; Mfn1 mRNA↑, Mfn2 mRNA↑	(Wang et al. 2019)
		Melatonin	The left anterior descending (LAD)coronary artery was ligation in C57BL/6 mice; primary cardiomyocytes treated with hypoxia reoxygenation	Promotes fusion, inhibits fission	Drp1↓, Fis1↓, Mff↓; OPA1↑	(Li et al. 2018)
MG132		Langendorff‐Perfused SD rats Heart IR Model; primary neonate rat myocardiocytes treated with hypoxia reoxygenation	Promotes fusion	Mfn2↑	(Song, Mihara, et al. 2015)
	
		Vitamin D3	The left anterior descending (LAD)coronary artery was ligation in C57BL/6 mice; H9C2 cells treated with hypoxia reoxygenation	Inhibits the translocation of Drp1	p‐Drp1↓, Mff↓	(Gao et al. 2012)
		Choline	The left anterior descending (LAD)coronary artery was ligation in C57BL/6 mice	Promotes fusion, inhibits fission	Drp1↓; Mfn2↑	(Gallo et al. 2024)
Therapeutic hypothermia	Langendorff‐Perfused Wistar rats Heart IR Model	blocked dephosphorylation of Drp1 S637 and mitochondrial translocation of Drp1	p‐Drp1^S637^/Drp1↑, p‐Drp1^S637^↑, Mito‐Drp1↓	(Jiang et al. 2014)
Calcific aortic valve disease	Others	MSI‐1436 (ptpl6 inhibitor)	Primary human VICs to induce osteogenic differentiation of VICs, (b‐glycerophosphate acid (b‐GA), dexamethasone, and ascorbic acid (b‐GA system) or Na2HPO4 were added to complete medium for 14 days. VICs were incubated in medium with recombinant human TGFb for 7 days)	NORMALIZES the mitochondrial fusion	L‐Opa1↓, S‐Opa1↑	(Xie et al. 2024)
Heart failure	A mitochondria‐targeted antioxidant	Mitoquinone (MitoQ)	The C57BL/6J mice ascending aortic constriction (AAC) was produced by placing a calibrated (31‐gauge needle) titanium clip on the ascending aorta.	Promotes fusion	Mfn2↑, L‐Opa1↑	(Yan et al. 2023)
	Anti‐hypertensive drug	Captopril	Wistar rats were treated with isoproterenol hydrochloride	Promotes fusion, inhibits fusion	Drp1↓; Mfn2↑	(C. Chen et al. 2017)
	Exercise	Exercise	Ischemic heart failure after LAD myocardial infarction ligation in Wistar Rats	Inhibits fusion	Mfn1↓, Mfn2↓	(Osellame et al. 2016)
	Others	Melatonin	Wistar rats were treated with isoproterenol hydrochloride	Promotes fusion, inhibits fusion	Drp1↓; Mfn2↑	(Chen et al. 2017)
		Melatonin+ captopril	Wistar rats were treated with isoproterenol hydrochloride	Promotes fusion, inhibits fusion	Drp1↓; Mfn2↑	(Chen et al. 2017)
Myocardial remodeling	Others	Melatonin	The left anterior descending (LAD)coronary artery was ligation in C57 mice	Promotes fusion	Mfn2↑	(Donnarumma et al. 2022)

## 6 Mitochondrial Dynamics in Age‐Related Brain Diseases

Age‐related neurological disorders, primarily PD and AD, are closely associated with aging‐mediated disruptions in the homeostasis of mitochondrial dynamics. Aging alters the expression of proteins involved in mitochondrial dynamics. Compared with 3‐month‐old mice, 12‐month‐old wild‐type (WT) mice exhibit reduced Mfn2 expression in the hippocampus (Ribeiro et al. 2019), with decreased expression of the mitochondrial fusion proteins Mfn1 and Mfn2 observed in both the hippocampus and cerebral cortex. In 12‐month‐old rats, Drp1 expression was increased in the substantia nigra, whereas Mfn1 and Opa1 expression was decreased (Yan et al. 2021). Similarly, Opa1 expression in the hippocampal tissue of 28‐month‐old mice was significantly lower than that in 2‐month‐old mice. Further studies revealed that knocking out Mfn2 in adult mice led to mitochondrial fragmentation in the hippocampus and cortex, ultimately causing neuronal death (Han et al. 2020). This age‐related trend of increased mitochondrial fission and reduced fusion may establish the pathological basis for the development of age‐related brain diseases, with the specific characteristics summarized in Table 3.

**TABLE 3:** Changes in mitochondrial dynamics in aging‐related brain diseases.

Disease	Model	Position	Protein changes in disease	Mitochondrial morphological changes	References
Alzheimer's disease	AD patients	Brain	SNO‐Drp1/Drp1↑	—	(Tang et al. 2014)
	AD patients	Brian	Cleavage fragment of Drp1↓, full‐length of Drp1↓	—	(Liu et al. 2021)
	AD patients	Brain	Drp1↑, Fis1↑; Mfn1↓, Mfn2↓, Opa1↓	—	(Yu et al. 2021)
	AD patients; oligodendrocytes treated with Aβ_1–42_O	Brain	Drp1 tetramer/Drp1↑; p‐Drp1^S616^↑	Extensive mitochondrial fragmentation	(Wu, Huang, et al. 2021)
	Aβ_1–42_ O were injected into the lateral ventricles of the cynomolgus macaques	Brain	Drp1↑; Mfn1↓, Opa1↓	—	(Kraus et al. 2021)
	Administering intracerebroventricular injection of Aβ_42_ to the SD rats; primary neural cells treated with Aβ_42_	Brain	p‐Drp1↑, MFF↑; Mfn1↓, Mfn2↓, Opa1↓	The length and area of mitochondria were significantly reduced	(Liu, Han, et al. 2024)
	Administering intracerebroventricular injection of Aβ_25–35_ to the C57BL/6J mice; PC12 cells treated with Aβ_25–35_	Brain	Drp1↑, Fis1↑; Mfn1↓, Mfn2↓, Opa1↓	—	(Atkins et al. 2016)
	Human iPSC‐derived astrocytes Human astrocytes treated with Aβ_42_ fibrils for 7 days and then cultured in Aβ42–free medium for 0, 6 or 12 days	Neurons	7d: p‐Drp1^S616^↑, 7d + 6d: p‐Drp1^S637^↑	The mitochondria were extremely long (7d + 6d); mitochondria profiles were very short, severely swollen and with disrupted cristae (7d + 12d)	(Chang et al. 2023)
	Platelets from AD patients were mixed with SH‐SY5Y cells previously depleted of endogenous mtDNA (ρ0 cells)	Neurons	Mito:Drp1↑, Fis1↑	—	(Wang and Song 2018)
	Primary cortical neurons treated with Aβ_25–35_O	Neurons	p‐Drp1^S616^/Drp1↑	A marked mitochondrial rupture, with circular mitochondria	(Li, Dang, et al. 2022)
	Primary neurons treated with Aβ_42_ O, SH‐SY5Y cells treated with Aβ_42_O	Neurons	Drp1↑; Mfn1↓, Mfn2↓, L‐Opa1↓	—	(Hasan et al. 2018)
	HT‐22 cells treated with Aβ_1–42_O	Neurons	Mito‐Drp1↑, Cyto:Drp1↓, p‐Drp1/Drp1↑	The number of punctate structures of Drp1 on mitochondria was increased	(Chan 2020)
	HT‐22 cells treated with Aβ_25–35_O	Neurons	Drp1↑, p‐Drp1/Drp1↓	The mitochondria accumulated damaged and divided fragments	(Wang, Liu, et al. 2011)
	HT‐22 cells treated with AβO	Neurons	p‐Drp1^S637^/Drp1↓	Shortening of mitochondrial length, increase in the damaged mitochondria fraction, increase in the number of mitochondria per cell	(Fu et al. 2021)
	HT22 cells treated with Aβ_1–42_	Neurons	Mfn2 mRNA↓	Most mitochondria were fragmented, the mitochondrial length was significantly shorter	(Quiles and Gustafsson 2022)
	SH‐SY5Y cells treated with Aβ_42_O	Neurons	Drp1↑, Fis1↑; Mfn1↓, Mfn2↓	—	(Yang, Yu, et al. 2017)
	SH‐SY5Y cells treated with Aβ_42_O	Neurons	Drp1↑; Mfn1↓, Mfn2↓, Opa1↓	Increased mitochondrial volume	(Gao and Hu 2021)
	SH‐SY5Y cells treated with Aβ_1–42_O	Neurons	Drp1↑, Fis1↑, MFF↑, p‐Drp1^S616^/p‐Drp1^S637^; Mfn1↓, Mfn2↓, Opa1↓	Mitochondria was extensively fragmented	(Wu, Huang, et al. 2021)
	PC12 cells treated with Aβ_25–35_	Neurons	Drp1↑, p‐Drp1^S616^↑, Fis1↑; Mfn1↓, Mfn2↓, Opa1↓	A large accumulation of damaged mitochondrial fragments with excessive fission and fusion, unclearly arranged crests, and defective organelle morphology	(Zhang et al. 2018)
	PC12 cells treated with Aβ_1–42_	Neurons	Opa1↓	—	(Ji et al. 2022)
	Neural Stem Cells treated with Aβ_1–42_O	Neurons	Mfn2↓	An effect on mitochondria integrity and function	(Qipshidze‐Kelm et al. 2014)
	Microglial BV2s treated with Aβ_1–42_O	Neurons	Mfn2 mRNA↓	—	(Hong et al. 2017)
	PD patients; SH‐SY5Y cells treated with MPP+	Brian	L‐Opa1↓, S‐Opa1↓	Mitochondria appeared more swollen and irregular in PD neurons, and cristae appeared to be deranged	(Bassiouni et al. 2023)
Parkinson's disease	PD patients	Brain (substantia nigra pars compacta)	Drp1↓	—	(Fang et al. 2022)
	Idiopathic PD patients	Brain	Drp1↑	—	(Zhou, Wang, et al. 2019)
	Monkey treated with MPTP	Brain (substantia nigra)	Mito:Drp1↑; Opa1↓	A marked mitochondrial ultrastructural injury was observed with significant swelling of the mitochondrial matrix	(Li, Xu, et al. 2022)
	C57BL/6 mice treated with MPTP	Brain (substantia nigra & striatum)	Mito‐Drp1↑, Cyto‐Drp1↓	—	(Sun et al. 2016)
	C57BL/6 mice treated with MPTP; SH‐SY5Y cells treated with MPP+	Neurons	Drp1↑	Neurite mitochondrial index (total mitochondrial length/neurite length)↓	(Zhang et al. 2016)
	C57BL/6 mice treated with MPTP	Brain (striatum)	Drp1↑	—	(Xu et al. 2017)
	C57BL/6 mice treated with MPTP	Brian	p‐Drp1^S637^↓; Mfn2↓, Opa1↓	—	(Jang et al. 2018)

	C57BL/6 mice treated with MPTP; SH‐SY5Y cells treated with rotenone	Brain	Cyto‐Drp1↓, Mito‐Drp1↑	More small mitochondria	(Geng et al. 2019)
	C57BL/6 mice treated with MPTP	Brain (substantia nigra)	Drp1↑	—	(Lee et al. 2019)
	C57BL/6 mice treated with MPTP	Brain	Mito‐Drp1↑	Mitochondria were damaged by MPTP, which features a disrupted and swollen structure, vague mitochondrial cristae, and condensate matrix	(Wang et al. 2022)
	C57BL/6 mice treated with MPTP; SH‐SY5Y cells treated with MPP+	Neurons	Drp1↑, Fis1↑; Mfn1↓, Mfn2↓, Opa1↓, Mito:Drp1↑, p‐Drp1^S616^/Drp1 ↑	Severe mitochondrial fragmentation, The mitochondria in treated cells appeared punctate or dot‐like	(Yang et al. 2021)
	C57BL/6J mice treated with MPTP; primary cultured neural precursor cells treated with MPP+	Brain	Drp1 tetramer ↑, Drp1 monomer↑, Fis1↑; Mfn1↓, Mfn2↓, Opa1↓	—	(Lee et al. 2021)
	C57BL/6J mice treated with MPTP; SH‐SY5Y cells treated with MPP+/rotenone	Neurons	Mice:Mfn1↓, Mfn2↓, Cell:: p‐Drp1^S616^/Drp1↓; Mfn1↓, Mfn2↓, Opa1↓	Mitochondrial fragmentation (short separate tubes or swollen tubes), and those mitochondria exhibited a swollen form, sparse matrix and disrupted cristae	(Zhang et al. 2023)
	C57BL/6 mice treated with MPTP	Brain	p‐Drp1^S637^/Drp1 ↓, p‐Drp1^S616^/Drp1↑, Drp1↓; Mfn1↓, Mfn2↓, Opa1↓	—	(Mondal et al. 2023)
	Wistar rats treated with rotenone	Brain (striatum)	Drp1↑	—	(Rahimmi et al. 2015)
	Wistar rats treated with rotenone	Brain (substantia nigra and striatum)	Drp1↑	—	(Ebrahimi et al. 2017)
	SD rats treated with rotenone; PC12 cells treated with rotenone	Brian (striatum)	p‐Drp1↑, Fis1↓; Mfn2↓, Opa1↓	The striatal neurons in the Rot group had vacuolated and swollen mitochondria with broken and disarrangement cristae	(Peng et al. 2018)
	SD rats treated with rotenone	Brain	Cyto:Drp1↓, Mito:Drp1↑	Large amounts of mitochondria in the rotenone group appeared to be vacuolated and swollen with incomplete and broken internal cristae	(Zhang, Huang, et al. 2020)
Wistar rats treated with 6‐OHDA	Brain	Drp1↑; Mfn2↓	—	(Anis et al. 2020)
	SD rats treated with 6‐OHDA	Brain (substantia nigra and striatum)	Drp1↓; Mfn2↓, L‐Opa1↓	—	(Chuang et al. 2017)
	C57BL/6J mice treated with α‐Syn PFF	Brain	Drp1↓	—	(Creed et al. 2022)
	SH‐SY5Y cells treated with different doses of MPP+	Neurons	Mito:Drp1↑; Mfn1↓, Mfn2↓	—	(Zhu et al. 2014)
	SH‐SY5Y cells treated with MPP+	Neurons	Drp1↑, Fis1↑; Mfn1↓, Mfn2↓, Opa1↓	Individual mitochondria disclosed a fragmented structure	(Gai et al. 2019)
	SH‐SY5Y cells treated with MPP+	Neurons	MFN1↓, MFN2↓, OPA1↓, Drp1↑, Fis1↑	—	(Ma, Li, et al. 2020)
	SH‐SY5Y cells treated with MPP+	Neurons	p‐Drp1/Drp1↓	—	(Reudhabibadh et al. 2021)
	PC12 cells treated with MPP+	Neurons	Cyto:Drp1↓, Mito:Drp1↑	—	(Zhang et al. 2019)
	Primary rat cortical neuron treated with different doses of MPP+	Neurons	Mito Drp1↑	—	(Chuang et al. 2016)
	PC12 cells treated with Rotenone	Neuron	Drp1↓, p‐Drp1↑, Fis1↓; Mfn2↓, Opa1↓	The number of small ring mitochondrial fragments increased significantly, a decrease of mitochondrial length and area, disorganized structure of mitochondrial cristae	(Peng et al. 2017)
	HT22 cells treated with Rotenone	Neurons	p‐Drp1↑, Fis1↑; Mfn2↓	Mitochondria presented with swelling and took on fine, granular shapes	(Li et al. 2017)
	SH‐SY5Y cells treated with Rotenone	Neurons	p‐Drp1^S616^↑, p‐Drp1^S637^↓; p‐Drp1^S616^/t‐Drp1↑; p‐Drp1^S637^/t‐Drp1↓; Mfn1, Mfn2, Opa1↓	—	(Ramalingam et al. 2023)
	N4741 cells derived from substantia nigra of mouse embryos treated with 6‐OHDA	Neurons	Drp1↑; Mfn1↓, Mfn2↓, Opa1↓	—	(Xi et al. 2018)
	SH‐SY5Y cells treated with 6‐OHDA	Neurons	Drp1↑, Fis1↑; Mfn2↓, Opa1↓	—	(Lin et al. 2020)
	Middle cerebral artery occlusion in SD rats (MCAO); human brain microvascular endothelial cells treated with OGD	Brain	Mfn2↓	The mitochondria were swollen, the mitochondrial cristae fracture disappeared	(Dong et al. 2019)
	Middle cerebral artery occlusion in SD rats (MCAO); PC12 cells treated with OGD	Brain	M1h:Drp↓, mtDrp↑M3, 6, 12, 24 h:Drp↑, mtDrp↓	M3h: the number of mitochondria of large diameter increased	(Zuo et al. 2014)
Cerebral infraction	Middle cerebral artery occlusion in SD rats (MCAO); human brain microvascular endothelial cells treated with OGD	Brain	Mito:p‐Drp1^S637^/Drp1↓	Vacuolar mitochondria increased and prominent cristae disappeared	(Zhou et al. 2021)
	Middle cerebral artery occlusion in C57BL/6J mice (MCAO); Primary cortical neurons treated with OGD	Brain	p‐Drp1/Drp1↑, Mff↑; Mfn1↓, Mfn2↓	Punctate and shorter mitochondria	(Wen et al. 2022)
	Bilateral common carotid artery occlusion (BCCAO) in SD rats	Brian (hippocampus)	Drp1↑, Fis1↑; Mfn2↓, Mfn1↓	Mitochondrial swelling, fragmentation, matrix disruption, and compromised or absent cristae	(Chen, Yang, Wang, Chen, et al. 2024)
	Bilateral common carotid artery occlusion (BCCAO) in SD rats	Brain	Drp1↑	Swelling and broken or disappearing cristae	(Wang, Peng, et al. 2024)
	Distal middle cerebral artery occlusion (dMCAO) in C57BL/6J mice	Brain	Drp1 mRNA↑	—	(Zhang, Wang, et al. 2024)
	A four‐vessel occlusion (4‐VO) model in SD rats	Brain	Cyto‐Drp1↓, Mito‐Drp1↑	Numerous autophagosomes enclosing mitochondria	(Zuo et al. 2016)
	Bilateral common carotid artery stenosis (BCAS) in C57BL/6J mice	Brain	p‐Drp1^S637^↓	misshapen mitochondria with disrupted internal structure	(Du et al. 2024)
	Primary cortical neurons treated with OGD	Neurons	p‐Drp1^S637^/Drp1↓	—	(Wu et al. 2017)
	Primary cortical neurons treated with OGD	Neurons	Drp1↑, Mfn1↓	—	(Sisalli et al. 2020)
	PC12 cells treated with OGD	Neurons	Drp1↑, Fis1↑; Mfn1↓, Mfn2↓	Mitochondria to shorten and mitochondrial cristae to appear coarse and ruptured	(Chen et al. 2018)
	Middle cerebral artery occlusion and reperfusion in SD rats (MCAO)	Brain	Cyto: Drp1↓, Mito:Drp1↑	—	(Feng et al. 2018)
	Middle cerebral artery occlusion and reperfusion in C57BL/6J mice (MCAO); primary microglia cells were treated with OGDR	Brain	Cyto: p‐Drp1^S616^/Drp1 ↓, Mito:Drp1↑	—	(Zhou, Chen, et al. 2019)
Cerebral ischemia reperfusion injury	Middle cerebral artery occlusion and reperfusion in C57BL/6J mice (MCAO)	Brain	Drp1↑, Fis1↓; Mfn2↓Opa1↓	Mitochondrial swelling and disarrayed cristae	(Kumari et al. 2012)
	Middle cerebral artery occlusion and reperfusion in SD rats (MCAO); BV‐2 microglial cells treated with OGDR	Brain	Drp1↑	Punctate and shorter mitochondria	(Hu, Zeng, et al. 2020)
	Middle cerebral artery occlusion and reperfusion in SD rats (MCAO)	Brain	Drp1↑	—	(Zhang, He, et al. 2020)
	Middle cerebral artery occlusion and reperfusion in SD rats (MCAO); HT22 mouse hippocampal neuroblastoma cells were treated with OGDR	Brain	Drp1↑, Fis1↑, Mff↑	—	(Yang et al. 2020)
	Middle cerebral artery occlusion and reperfusion in SD rats (MCAO)	Brain	Fis1↑	Disappearance of bilayer membrane structure, vacuolar degeneration and swelling, and loss of cristae	(Tang et al. 2020)
	Middle cerebral artery occlusion and reperfusion in C57 mice (MCAO)	Brain	Cyto:Drp1↓, p‐Drp1^S616^/Drp1↑, Mito:Drp1↑	Mitochondrial length was decrease, cristae densities were reduced	(Zeng et al. 2022)
	Middle cerebral artery occlusion and reperfusion in SD rats (MCAO); HT22 cells treated with OGDR	Brain	Mfn2↓	The degree of mitochondrial fragmentation increased and the area of mitochondria decreased	(Xu et al. 2023)
	Middle cerebral artery occlusion and reperfusion in C57BL/6J mice (MCAO)	Brain	Fis1↑; Mfn1↓, Mfn2↓, Opa1↓	Mitochondria were swollen, the structure was damaged, the mitochondrial membrane was ruptured as well as irregular stretching of mitochondria and mitochondrial fission both increased	(Tang et al. 2023)
	Middle cerebral artery occlusion and reperfusion in C57BL/6J mice (MCAO)	Brain	Drp1↑, Fis1↑; Mfn2↓, Opa1↓	Extensive mitochondrial fission and fragmentation were observed	(Liu, Zhao, et al. 2023)
	Middle cerebral artery occlusion and reperfusion in SD rats (MCAO)	Brain	Mito:Drp1↑, Fis1↑; Mfn1↓, Mfn2↓, Opa1↓	—	(Du et al. 2023)
	Middle cerebral artery occlusion and reperfusion in SD rats (MCAO)	Brain	Fis1↑; Mfn2↓, Opa1↓	Notable mitochondrial fission and fragmentation	(Ge et al. 2024)
	Middle cerebral artery occlusion and reperfusion in SD rats (MCAO)	Brain	Drp1↑, Fis1↑, Mff↑; Opa1↓, Mfn1↓, Mfn2↓	Mitochondria exhibited severe swelling, increased volume, matrix lysis, cristae disappearance, and vacuolization	(Chen, Yang, Zhou, Yu, et al. 2024)

	Middle cerebral artery occlusion and reperfusion in C57BL/6 mice (MCAO)	Brain	p‐Drp1^S616^↑, Mito:Drp1↑	Injured and cracked mitochondrial cristae and shortened mitochondrial length in neurons surrounding ischemic areas	(Zhang and Gong 2024)
	Middle cerebral artery occlusion and reperfusion in C57BL/6 mice (MCAO); HT22 cells treated with OGDR	Brain	Mfn2↓	—	(Xu et al. 2025)
	Middle cerebral artery occlusion and reperfusion in SD rats (MCAO); PC12 cells treated with OGDR	Brain	Drp1↑; Mfn1↓, Mfn2↓	Excessive mitochondrial swelling with vague cristae	(Chen, Duan, Zou, Yang, et al. 2024)
	Middle cerebral artery occlusion and reperfusion in C57BL/6 mice (MCAO)	Brain	Mfn1↓, Mfn2↓, Opa1↓	A shorter mean mitochondrial length	(Zhu et al. 2024)
	Transient middle cerebral artery occlusion/reperfusion (tMCAO/R) Wistar rats	Brain	Cyto‐Drp1↓, Mito‐Drp1↑	—	(Ali et al. 2022)
	Bilateral common carotid artery occlusion (BCCAO) and reperfusion in ICR mice	Brian	Mfn2↓, Opa1↓	—	(Ning et al. 2024)
	A four‐vessel occlusion and reperfusion (4‐VO) in Wistar rats	Brain	p‐Drp1^S637^↓, Mito:Drp1↑	——	(Zhan et al. 2019)
	Left middle cerebral artery occlusion and reperfusion in SD rats (LMCA)	Brain	Drp1↑	——	(Chen et al. 2021)
	HT22 mouse hippocampal neuroblastoma cells treated with OGDR	Neurons	Drp1↑; Mfn2↓, Opa1↓	The mitochondria cristae were irregular, dilated, and without a parallel distribution	(Nasoni et al. 2021)
	HT22 mouse hippocampal neuroblastoma cells treated with OGDR	Neurons	Drp1↑, Fis1↑	Some mitochondria were divided and engulfed by vacuolar structures	(Wu et al. 2022)
	HT22 cells treated with OGDR	Neurons	Mfn2↓	—	(Li et al. 2024)
	PC12 cells treated with OGDR	Neurons	Mfn2↓	—	(Zeng et al. 2021)
	Primary cortical neurons treated with OGDR	Neurons	R3h:Drp1↓; Mfn2↓, Opa1↓, R24h:Mfn2↓	—	(Wojtyniak et al. 2022)
Primary hippocampal neurons treated with OGDR	Neurons	p‐Drp1^S637^↓; Mfn2↓, Mfn1↓, Opa1↓	Swollen mitochondria with decreased ridge density, and ruptured membranes	(Yang et al. 2024)

### 6.1 AD

The brain is a high‐energy‐demanding organ, with neuronal electrophysiological activity dependent on mitochondrial OXPHOS for energy supply (Lin and Beal 2006). Mitochondria also regulate ROS production and mediate fusion and fission (Burtscher et al. 2023), with their kinetic homeostasis being central to neuronal function (Osellame et al. 2016). Mitochondrial dysfunction is a key driver of the AD pathological cascade, inducing β‐amyloid (Aβ) accumulation, upregulating phosphorylated tau, triggering oxidative damage, and ultimately causing neuronal injury (Wang et al. 2008). These pathological processes are driven by the hyperactivation of Drp1. Aβ activates a cascade of kinases, including cyclin‐dependent kinase 5 (CDK5) and glycogen synthase kinase 3 beta (GSK3β), which specifically induces phosphorylation at the S616 site of Drp1, promoting its tetramerization and translocation to the mitochondria, thereby driving pathological fission. GSK3β also indirectly regulates CDK5 activity, sustaining the amplification of fission signals (Rong et al. 2020). Recent studies have reported that the modification of interferon‐stimulated gene 15 (ISG15), as a novel P, can act synergistically with S616 phosphorylation to increase Drp1 fission activity and disrupt mtDNA stability, providing a new direction for investigating the abnormal amplification of Drp1 function. Concurrently, calpain‐mediated proteolytic cleavage of Drp1 and S‐nitrosylation (SNO‐Drp1) collectively constitute a multipronged attack network, leading to the complete collapse of the mitochondrial quality control system (Cho et al. 2009; Jiang et al. 2019).

Specifically, in the frontal cortex of AD patients, Drp1 expression is reduced, whereas Fis1 expression is increased. Moreover, the levels of Opa1, Mfn1, and Mfn2 are decreased, and the Drp1 tetramer/total Drp1 ratio, p‐Drp1S616 level, and SNO‐Drp1/total Drp1 ratio are significantly increased. Furthermore, calpain activation in AD significantly reduces the levels of full‐length Drp1 and its lysosomal fragments (Jiang et al. 2019), disrupting mitochondrial dynamic homeostasis and accelerating disease progression. Peripheral blood lymphocyte analysis revealed that compared with controls, AD patients had increased SNO‐Drp1 and Fis1 expression but decreased Drp1 expression. In differentiated cell lines containing mtDNA from sporadic AD patients, the levels of mitotic‐related proteins such as Drp1 and Fis1 also tended to increase, confirming the prevalence of disrupted mitochondrial dynamics in patients with AD (Silva et al. 2017; Wang, Song, et al. 2012). In addition to these common pathological backgrounds, different subtypes of neurons exhibit distinct responses.

The effect of Aβ treatment on Drp1 expression in neuronal cell lines is cell specific. Cytoplasmic Drp1 levels increase in some cell lines (Ahmed et al. 2019; Bartolome et al. 2018; Cheng et al. 2021; Kuruva et al. 2017; Lee et al. 2018) but decrease in others (Lee et al. 2018). The p‐Drp1/total Drp1 ratio may increase or decrease but is always accompanied by a decrease in the p‐Drp1S637/total Drp1 ratio, an increase in the p‐Drp1S616/p‐Drp1S637 ratio, and increased Fis1 and Mff expression, along with decreased Mfn1, Mfn2, and Opa1 (including L‐Opa1) expression (Ahmed et al. 2019; Ayabe et al. 2022; Bartolome et al. 2018; Cheng et al. 2021; de la Cueva et al. 2022; Kang et al. 2018; Kuruva et al. 2017; Lee et al. 2018). Furthermore, the effects of Aβ1‐42 on distinct neuronal subtypes differ: treatment with neural stem cells and BV2 microglia reduces Mfn2 expression (Cieślik et al. 2020; Ribeiro et al. 2019), increases the ratio of the Drp1 tetramer to total Drp1, increases the p‐Drp1S616 level in oligodendrocytes, decreases the p‐Drp1S637/total Drp1 ratio, and decreases Mfn2 and Opa1 expression, whereas treatment with Aβ significantly increases the number of Drp1 aggregates in astrocytes (Kim et al. 2022). Notably, Opa1 overexpression mitigates Aβ1‐42‐induced mitochondrial dysfunction and neuronal apoptosis (Dowding et al. 2014), providing experimental evidence for intervention strategies targeting mitochondrial fusion proteins. These cell‐specific observations suggest that the extreme sensitivity of neurons to energy fluctuations may increase their susceptibility to acute mitochondrial damage mediated by S616 phosphorylation, whereas alterations in mitochondrial dynamics in glial cells may play a greater role in maintaining the neuroinflammatory microenvironment and impairing Aβ clearance.

Traditional broad‐spectrum antioxidant or single‐protein modulation strategies are insufficient to counteract these complex mechanisms. Current drug development must shift from focusing on treating phenotypes to blocking specific pathogenic interactions while simultaneously employing multitarget synergistic regulation strategies. Allosteric modulation to block the pathogenic interaction between Drp1 and Aβ is among the most promising approaches. Diethyl (3,4‐dihydroxyphenethylamino) (quinolin‐4‐yl) methylphosphonic acid (DDQ) effectively inhibits abnormal Aβ–Drp1 interactions, providing neuroprotective effects for neurons in AD (Kuruva et al. 2017). Among natural product interventions, *Cornus officinalis* extract and its active components can correct mitochondrial fission/fusion imbalance and promote mitophagy via the PTEN‐induced kinase (PINK1)/parkin RBR E3 ubiquitin protein ligase (Parkin) pathway, thereby inhibiting the assembly and activation of the NLRP3 inflammasome and reducing IL‐1β release (Cao et al. 2024; F. Zhou et al. 2025; Zhou et al. 2023). Furthermore, the active components of *Cornus officinalis* have been shown to modulate astrocyte phenotypes via the protein kinase B (AKT)/nuclear factor erythroid 2‐related factor 2 (Nrf2)/nuclear factor kappa‐light‐chain‐enhancer of activated B cells (NF‐κB) signaling pathway, inducing their conversion to the neuroprotective A2 phenotype (Shi et al. 2022). Studies indicate that enhancing PINK1/Parkin‐mediated mitophagy is among the key mechanisms for inhibiting NLRP3 inflammasome activation (Zhang, Du, et al. 2022), whereas promoting the polarization of astrocytes toward the A2 phenotype is an important strategy for alleviating neuroinflammation and secondary damage to the central nervous system (Ma, Li, et al. 2020; Wang et al. 2021). Given that mitochondrial‐targeted delivery can increase the bioavailability of natural products, the XJB and magnolol conjugates prepared using mitochondrial‐targeting peptide XJB conjugation technology can specifically accumulate in the mitochondrial matrix and efficiently activate the deacetylase SIRT3, thereby increasing Mfn1 expression and initiating mitophagy; their neuroprotective efficacy is significantly superior to that of the free drug, and they exhibit lower toxicity (Shan et al. 2019).

**FIGURE 5:**
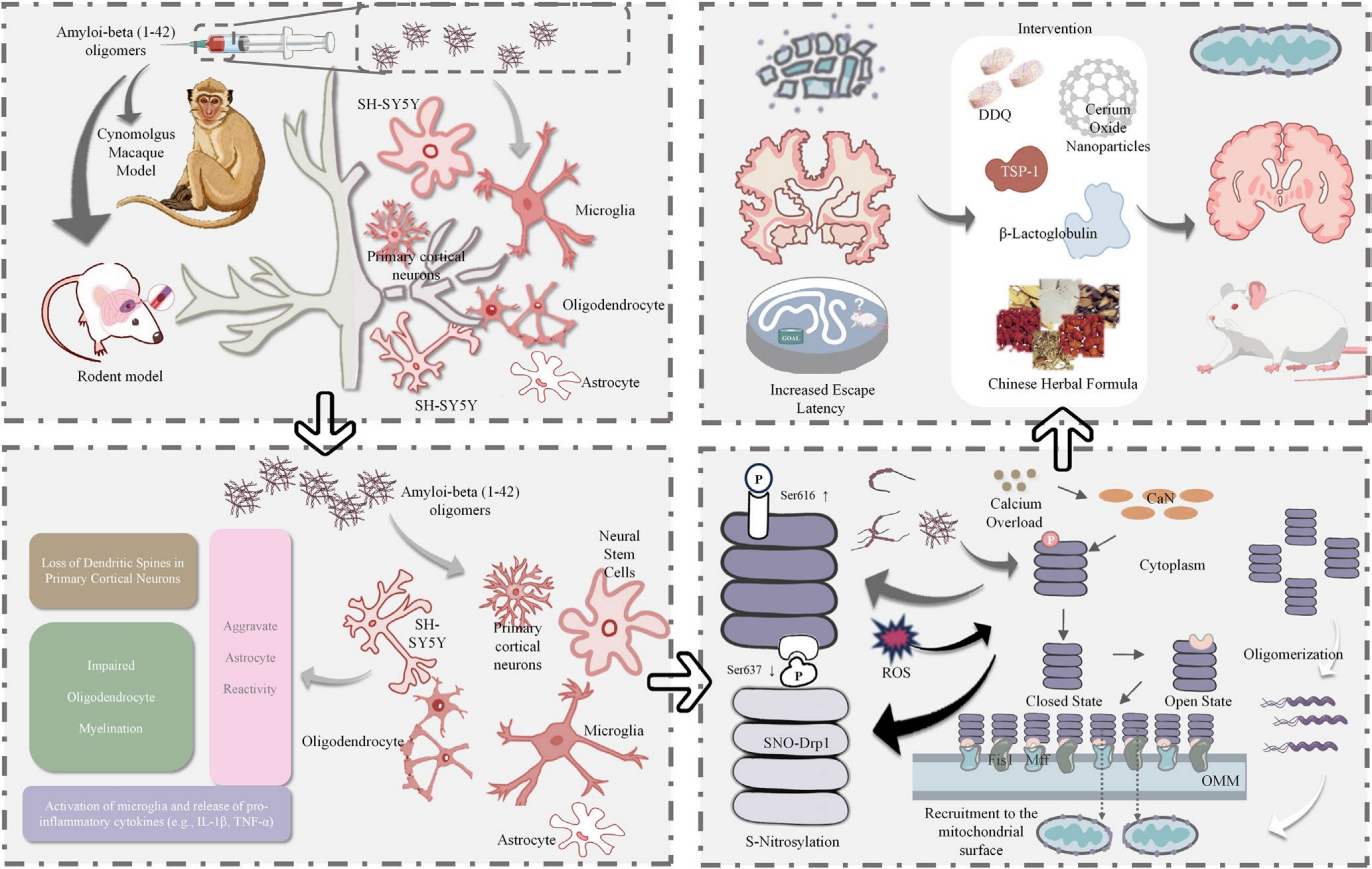
Schematic illustration of mitochondrial dynamics dysregulation in alzheimer's disease: Aβ models, cellular effects, and therapeutic interventions. This schematic elucidates mitochondrial dynamics dysregulation in AD driven by amyloid‐beta oligomers. The top‐left quadrant depicts AD models, including Cynomolgus Macaque, rodent, and cellular models such as SH‐SY5Y cells, primary cortical neurons, microglia, oligodendrocytes and astrocytes, all exposed to Aβ_1_₋_42_ oligomers. The bottom‐left illustrates Aβ‐induced cellular deficits: loss of dendritic spines, impaired oligodendrocyte myelination, activated microglia releasing inflammatory cytokines, and aggravated astrocyte reactivity. The bottom‐right details molecular events: calcium overload activates calcineurin, promoting Drp1 phosphorylation and S‐nitrosylated Drp1, which leads to Drp1 oligomerization on the outer mitochondrial membrane via receptors and enhanced fission. The top‐right presents interventions: DDQ, cerium oxide nanoparticles, thrombospondin‐1, β‐lactoglobulin, and Chinese herbal formulas. These interventions restore mitochondrial homeostasis and improve cognitive deficits. Collectively, it depicts the Aβ‐driven mitochondrial dynamics imbalance and therapeutic strategies in AD.

In summary, during the progression of AD, Aβ accumulation induces excessive Drp1 activation through multiple molecular mechanisms. Combined with various posttranslational modifications, this leads to a cascade of damage, resulting in excessive mitochondrial fission and impaired fusion function. This characteristic has been validated in not only brain tissue and peripheral blood lymphocytes from AD patients but also various cellular models. Various neuronal cell subtypes exhibit significantly different responses to Aβ stimulation, with distinct phenotypic and functional effects of altered mitochondrial dynamics, which also explains the specialized roles of different cells in the pathology of AD. The aforementioned Aβ‐induced mitochondrial kinetic disorders and intervention mechanisms are visually represented in Figure 5, which systematically integrates the relationships among AD models, cellular effects, and therapeutic strategies. Current interventions targeting this pathway include the use of novel small molecules, natural bioactive compounds, and mitochondrial‐targeted delivery technologies. These approaches aim to specifically block abnormal Aβ–Drp1 interactions, balance mitochondrial dynamics, and regulate inflammation and cellular phenotypes. However, all related studies are currently in the preclinical exploration phase, and no systematic clinical intervention trials have been conducted.

### 6.2 PD

PD is the second most common neurodegenerative disorder and is characterized by the progressive degeneration of substantia nigra neurons and the abnormal accumulation of α‐synuclein (α‐Syn) (Ahmed et al. 2019). While its exact etiology remains incompletely understood, mitochondrial dysfunction is recognized as a central pathogenic hub involving multifactorial mechanisms, including genetic inheritance, aging, and environmental factors. Disruption of the equilibrium of mitochondrial dynamics is one of the key pathways through which mitochondrial dysfunction exerts its effects in PD. This process is not only present in sporadic PD but also plays core regulatory roles across various PD experimental models.

In patients with sporadic PD, disruption of mitochondrial dynamics manifests primarily as the abnormal expression of key regulatory proteins. Cleavage of the long OPA1 isoform may partially contribute to mitochondrial fragmentation (Creed et al. 2022; Santos et al. 2015). Additionally, elevated phosphorylation of Drp1 promotes its transport to mitochondria, potentially accelerating mitochondrial fission (Creed et al. 2022). Drp1 expression is elevated in the substantia nigra of idiopathic PD patients, while no significant differences in Mfn1, Mfn2, Opa1, or Mff expression have been observed (Zhao et al. 2017). Conversely, another study reported reduced Drp1 expression in the substantia nigra pars compacta (SNpc) of PD patients, leading to elevated intracellular Ca^2+^ levels, excessive glutamate release, and increased synaptic excitotoxicity (Hoekstra et al. 2015). Concurrently, Drp1 expression levels tended to decrease in peripheral blood lymphocytes from PD patients. Mfn2 is a key regulator of mitochondrial network homeostasis in midbrain dopaminergic neurons. Mfn2 knockout in mice results in severe and progressive mitochondrial dysfunction in dopaminergic neurons, leading to neurodegeneration and PD‐like symptoms (Filograna et al. 2021).

Currently, the experimental models used to study PD can be divided into two main types: toxin‐induced models and α‐Syn prefibrillar models. Changes in mitochondrial dynamics across these different models share common characteristics but also differ. 1‐Methyl‐4‐phenyl‐1,2,6‐tetrahydropyridine (MPTP) and its active metabolite 1‐methyl‐4‐phenylpyridinium ion (MPP^+^) exhibit potent toxicity toward dopaminergic neurons and are commonly used to establish experimental models of PD (Gomez‐Lazaro et al. 2008). 6‐Hydroxydopamine (Iravanpour et al. 2021) and rotenone (Rahimmi et al. 2015) have also been used to establish animal and cellular models of PD. A common feature of these models is increased mitochondrial fission and reduced fusion. For example, in nonhuman primate models of PD established by MPTP injection, Drp1 expression is increased in the substantia nigra mitochondria, whereas Opa1 expression is significantly reduced (Chen, Zhu, Yuan, Ma, et al. 2024). Drp1 expression is selectively upregulated in vulnerable neurons. In vivo, Drp1 is strongly activated in brain tissue, accompanied by decreased p‐Drp1S637 levels and decreased expression of Opa1 and Mfn2 (Filichia et al. 2016; Irrcher et al. 2010; Mondal et al. 2023; Rappold et al. 2014; Zhao et al. 2021). Mfn2 overexpression partially alleviates the MPTP‐induced reduction in striatal dopaminergic neuronal fiber density and decreases dopamine concentrations while nearly completely blocking the significant MPTP‐induced loss of substantia nigra dopaminergic neurons (Zhao et al. 2021).

**FIGURE 6:**
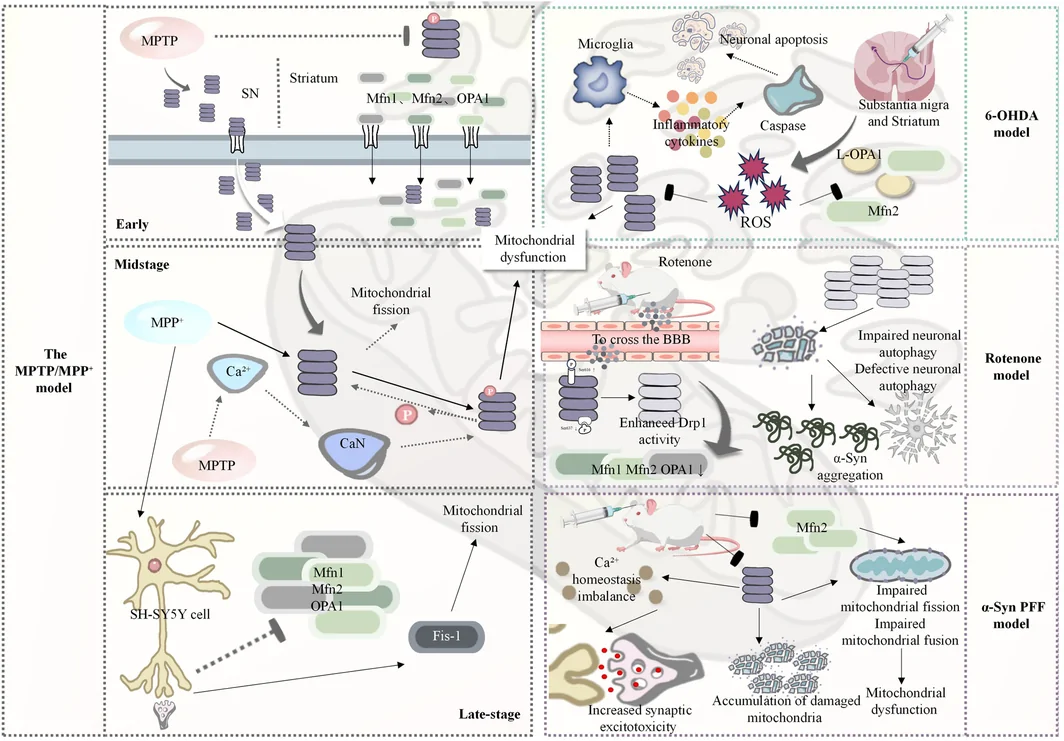
Schematic illustration of mitochondrial dynamics dysregulation in PD models: MPTP/MPP^+^, 6‐OHDA, rotenone, and α‐Syn PFF. This schematic outlines mitochondrial dynamics dysregulation across major PD models. In the MPTP/MPP^+^ model, MPTP metabolizes to MPP^+^, triggering Ca^2+^ overload and CaN activation, which promotes Drp1‐mediated mitochondrial fission while downregulating fusion proteins, leading to mitochondrial dysfunction. The 6‐OHDA model demonstrates Drp1 activation, ROS overproduction, and reduced L‐OPA1/Mfn2, initiating microglial activation, inflammatory cytokines release, and neuronal apoptosis. The rotenone model depicts enhanced Drp1 activity, impaired neuronal autophagy, and α‐synuclein aggregation after crossing the blood–brain barrier (BBB). The α‐Syn PFF model exhibits Ca^2+^ homeostasis imbalance and impaired mitochondrial fusion, resulting in increased synaptic excitotoxicity and accumulation of damaged mitochondria. Collectively, these models illustrate how mitochondrial dynamics imbalance drives PD pathogenesis across genetic, toxic, and protein aggregation contexts.

MPP^+^ treatment of dopaminergic neurons promotes the translocation of Drp1 to mitochondria, thereby triggering mitochondrial fission (Gomez‐Lazaro et al. 2008; Wang, Su, et al. 2011). Concurrently, MPP^+^ treatment increases the dephosphorylation level at the Drp1 Ser656 site, which further exacerbates MPP^+^‐induced mitochondrial dysfunction and increases apoptosis rates (Wang, Su, et al. 2011). In cortical neurons exposed to MPP^+^, increased Drp1 expression can be detected within 4 h (Chuang et al. 2016). 6‐Hydroxydopamine (6‐OHDA) can also be used to establish animal or cellular models of PD. Analyses of different brain regions in 6‐OHDA‐induced animal models of PD revealed decreased levels of L‐Opa1 and Mfn2 in the striatum and substantia nigra, as well as reduced Drp1 expression (Iravanpour et al. 2021). However, another study reported increased Drp1 expression in the substantia nigra (Zhu et al. 2014). In in vitro rotenone models, elevated p‐Drp1S616 levels and reduced p‐Drp1S637 levels resulted in increased p‐Drp1S616/total Drp1 ratios and decreased p‐Drp1S637/total Drp1 ratios, accompanied by reduced Mfn1, Mfn2, and Opa1 expression (Peng et al. 2017; Rahimmi et al. 2015; Ramalingam et al. 2023; Zhang, Huang, et al. 2020). However, classic PD models induced by toxins such as 6‐OHDA and MPTP have inherent limitations. They fail to effectively mimic the protein misfolding and aggregation processes that cause cellular dysfunction, synaptic loss, and brain damage. Furthermore, they cannot reproduce PD–associated molecular pathological features, such as α‐Syn aggregation and Lewy body formation. Rose B et al. established a model that more closely resembled clinical PD by injecting small oligomers (< 30 nm) of preformed fibrils (PFF) into the mouse striatum, and Drp1 expression levels were reduced in this model (Creed et al. 2022). These findings underscore the potential role of mitochondrial dynamics‐associated proteins in key pathological processes of PD, including α‐Syn aggregation and Lewy body formation. The specific mechanisms underlying mitochondrial dysregulation in the aforementioned multimodel systems are shown in Figure 6, which systematically integrates molecular events with pathological phenotypes across different PD models.

Intervention strategies based on mitochondrial dynamics have demonstrated neuroprotective effects in various PD models. First, some strategies regulate mitochondrial fission and biogenesis simultaneously. Mdivi‐1 inhibits mitochondrial fission and promotes the nuclear translocation of PGC‐1α by activating the CaMKII/CREB pathway, thereby upregulating the expression of downstream NRF1 and TFAM and initiating mitochondrial biogenesis (Ma, Yang, et al. 2025). Mitochondrial renewal contributes to neuroprotection; inhibiting mitochondrial fission alone leads to the accumulation of aged mitochondria. When evaluating the efficacy of relevant drugs, considering only mitochondrial morphological indicators is insufficient; the interplay between mitochondrial fission and biogenesis must also be considered. Other fission inhibitors, such as FLZ and selective peptide inhibitors, such as P110, reduce dopaminergic neuron loss and synaptic damage through similar mechanisms (Filichia et al. 2016; Qi et al. 2013; Yang et al. 2021).

Targeting mitochondrial quality control checkpoints represents another important approach for PD intervention, in which the coordinated regulation of mitochondrial dynamics and autophagy plays a crucial role. Studies have shown that the lncRNA NR_030777 can simultaneously regulate CDK1‐dependent Drp1 phosphorylation and ATG12‐ATG5 complex formation; moderate manganese supplementation can increase mitophagy and restore dynamic homeostasis via the PINK1/Parkin pathway, and these protective effects depend on moderate mitochondrial fission (Deng et al. 2008; Yu et al. 2011). Genetic studies have also confirmed that the PINK1/Parkin pathway maintains mitochondrial integrity in dopaminergic neurons by promoting fission or inhibiting fusion. On the basis of this coupling mechanism, various mitochondria‐targeting compounds have demonstrated clear neuroprotective effects in PD models. For example, in a 6‐OHDA‐induced animal model of PD, alpha‐lipoamide not only restored midbrain ATP levels but also significantly improved mitochondrial fragmentation and vacuolization by regulating the balance of mitochondrial fission, fusion, and transport protein expression, thereby exerting neuroprotective effects (Moradi Vastegani et al. 2023). In addition, the LRRK2 inhibitor GSK2578215A has been shown to act as a precursor to autophagy initiation via Drp1‐mediated mitochondrial fission, with mitochondria‐derived ROS used as second messengers to trigger protective autophagy for the clearance of damaged mitochondria; this process relies on the inhibition of fission rather than fusion (Zheng et al. 2024). Furthermore, the natural flavonoid kaempferol was shown to exhibit specific neuroprotective effects in a rotenone toxicity model. Its mechanism involves increasing the rate of mitochondrial fission and the number of autophagosomes containing mitochondria; this protective mechanism, mediated by enhanced autophagy, can be blocked by autophagy inhibitors, further validating the functional coupling between kinetic remodeling and autophagic clearance (Ahn et al. 2024). Moreover, the novel peptide inhibitor P110 suppresses pathological hyperfission by selectively blocking the interaction between Drp1 and Fis1; P110 reduces ROS production, improves mitochondrial integrity, and decreases autophagic death in dopaminergic neurons without affecting baseline physiological fission (Han et al. 2024). Collectively, these studies indicate that precise interventions aimed at the synergistic interface between mitochondrial fission and autophagy represent effective therapeutic strategies for PD.

Given the complexity of PD pathology, single‐target small molecules face translational bottlenecks, whereas synergistic interventions targeting multiple pathways demonstrate superior performance in terms of restoring homeostasis. Multiple traditional Chinese medicine components exert anti‐Parkinsonian effects by regulating mitochondrial dynamics. Strychnine glycosides, mangiferin and luteolin inhibit MPTP/MPP^+^‐induced mitochondrial fragmentation, thereby helping maintain mitochondrial homeostasis (Wang et al. 2022; Xu et al. 2017; Zhu, Sun, et al. 2019). Ferulic acid restores impaired mitochondrial dynamics by reducing mitochondrial Drp1 expression and regulating its downstream target Mfn2 (Anis et al. 2020). Additionally, salicylic acid protects neuronal cells from mitochondrial dysregulation in 6‐OHDA‐induced Parkinsonism (Lin et al. 2020). Harman (Ebrahimi et al. 2017) and andrographolide (Ebrahimi et al. 2017) counteract the disruption of mitochondrial dynamics in rotenone‐induced PD models. Traditional Chinese medicinal formulas also demonstrate potential. Dabu Yinwan (Gai et al. 2019), Qianzheng Powder (Gai et al. 2019), and Bu Yin Qian Zheng Formula (Ma, Gai, et al. 2020) protect cells from MPP^+^‐induced damage by balancing mitochondrial fission and fusion. With respect to physical stimulation therapies, deep brain stimulation of the subthalamic nucleus (Chen, Zhu, Yuan, Ma, et al. 2024) and anodal transcranial direct current stimulation exert neuroprotective effects by inhibiting mitochondrial fission, regulating mitochondrial kinetic homeostasis, and promoting mitochondrial fusion (Lee et al. 2019). Furthermore, endurance exercise alleviates MPTP‐induced damage in PD (Jang et al. 2018). Treadmill training increases dopaminergic neuronal activity and restores mitochondrial function in PD model rats. These effects may be mediated by increased mitochondrial turnover, which promotes mitochondrial fusion, fission, and clearance and ultimately leads to an increase in the number of mitochondria (Chuang et al. 2017).

Existing research confirms that mitochondrial dysregulation is involved in the pathological progression of PD. Findings from studies on sporadic PD are inconsistent, and the molecular phenotypes of various experimental models differ. Toxin models are characterized primarily by excessive mitochondrial fission and impaired fusion, whereas α‐Syn models exhibit distinct features; furthermore, classic toxin models have limitations in terms of accurately simulating disease pathology. Currently, various approaches, including the use of small‐molecule drugs, traditional Chinese medicine components, physical interventions, and exercise, have demonstrated efficacy to some extent; however, nearly all evidence stems from in vitro cellular experiments and rodent models, and clinical trial data with mitochondrial dynamics as the primary endpoint are lacking. In future research, the long‐term efficacy of candidate interventions in α‐Syn models that more closely mimic human PD pathology should be validated. Moreover, mitochondrial dynamics‐related biomarkers in peripheral blood or cerebrospinal fluid should be identified to support early clinical translation assessments. In addition, the safety of strategies targeting mitochondrial fission should be evaluated in animal models that incorporate comorbidities and aging factors.

### 6.3 Cerebral Infarction

Cerebral ischemia–hypoxia can disrupt mitochondrial homeostasis, and mitochondrial dysfunction directly mediates ischemic neuronal damage; this pathological feature is highly conserved across various in vivo and in vitro ischemia models. In a mouse model of middle cerebral artery occlusion (MCAO), analysis of brain tissue revealed elevated mitochondrial Drp1 expression, an increased p‐Drp1/Drp1 ratio, and a decreased p‐Drp1S637/Drp1 ratio. Furthermore, Fis1 and Mff expression increased, whereas Opa1, Mfn1, and Mfn2 expression significantly decreased. These findings suggest that inhibiting neuronal Drp1 dephosphorylation may help prevent cerebral ischemia (Lan et al. 2024; Wen et al. 2022). Consistent trends in protein expression were also observed in mice with bilateral common carotid artery occlusion and mice with bilateral common carotid artery stenosis (Chen, Yang, Wang, Chen, et al. 2024; Du et al. 2024; Wang, Peng, et al. 2024). In primary neurons, neuronal cell lines, and human brain microvascular endothelial cells subjected to oxygen–glucose deprivation, a similar pattern characterized by a decreased p‐Drp1S637/Drp1 ratio, increased cleaved protein expression, and decreased fusion protein expression was detected (Chen et al. 2018; Sisalli et al. 2020; Wen et al. 2022; Zhou et al. 2021).

The subcellular localization and functional state of Drp1 exhibit significant spatiotemporal heterogeneity. In a rat model of MCAO, acute fission occurred 1 h after ischemia because of the rapid accumulation of mitochondrial Drp1; 3–24 h after ischemia, cytoplasmic Drp1 levels increased, whereas mitochondrial Drp1 levels decreased, suggesting that sustained stress can disrupt the mitochondrial translocation mechanism of Drp1 (Zhou et al. 2021). In contrast, in a rat model of global cerebral ischemia (four‐vessel occlusion), a sustained decrease in cytoplasmic Drp1 levels and an increase in mitochondrial Drp1 levels were observed (Zuo et al. 2016). This difference reflects the distinct regulatory patterns of mitochondrial dynamics in focal and global ischemia and suggests that the therapeutic window should be selected according to the dynamic distribution characteristics of Drp1.

The functional activation of Drp1 depends on the synergistic regulation of posttranslational modifications. Ischemia increases neuronal AMPK activity, induces Drp1 phosphorylation, and subsequently promotes the interaction between GCN5‐like protein 1 (GCN5L1) and Drp1, mediating its acetylation and ultimately amplifying mitochondrial fission effects (Zhang, Wang, et al. 2024). The inhibition of AMPK blocks Drp1 acetylation and excessive fission, which induces neuroprotective effects. In addition to classic posttranslational modifications, the expression of the RNA‐binding protein RCAN1.1L is abnormally elevated in neurons in the ischemic penumbra. Stabilizing ATF2 mRNA levels promotes the accumulation of mitochondrial ATF2, which upregulates Fis1 expression and drives mitochondrial fission, thereby extending kinetic regulation to the posttranscriptional stage (Zuo et al. 2014). Furthermore, abnormal mitochondrial fission exacerbates damage by disrupting mitochondrial quality control. In the early stages of ischemia, the moderate activation of Drp1‐dependent mitophagy can result in the clearance of damaged mitochondria and promote cell survival, whereas persistent and excessive mitochondrial fission leads to mitochondrial dysfunction and triggers abnormal autophagic flux. For example, abnormal activation of Drp1 during the I/R period can result in the accumulation of autophagosomes, inducing inflammatory cascades (Zeng et al. 2022). Therefore, when mitochondrial dynamics are targeted for disease treatment, modulating autophagic flux may facilitate synergistic neural repair (Alzhrani et al. 2026; Zuo et al. 2020).

Multiple intervention strategies targeting these pathological mechanisms have demonstrated potential neuroprotective effects. Pentoxifylline is a commonly used clinical drug. In addition to its classic role in improving blood flow, new mechanisms of action have been identified in recent studies. Pentoxifylline specifically inhibits the GCN5‐like protein 1‐mediated acetylation of Drp1; mitochondrial turnover at the cellular level is not significantly affected, and ischemia‐induced mitochondrial damage is effectively alleviated (Zhang et al. 2025). Additionally, the novel PDE4 inhibitor ZX21011 does not act directly on the Drp1 protein; instead, it increases the phosphorylation level of serine 9 on GSK3β, reduces the binding capacity between GSK3β and Drp1, and decreases the phosphorylation level of serine 616 on Drp1 under pathological conditions (Wen et al. 2025). This process represents an indirect allosteric regulatory mechanism that circumvents the off‐target effects associated with directly targeting the Drp1 GTPase domain, reflecting the trend toward increasingly refined small‐molecule drug development. However, the aforementioned targeted interventions involving Drp1 and mitochondrial dynamics are currently within the preclinical research stage and have not yet been translated into mature clinical treatments. Although the mechanism through which coenzyme Q10, one of the few approved clinical drugs, improves mitochondrial function has been elucidated in basic research, it is still primarily considered a multitarget neuroprotective agent rather than a specific regulator of mitochondrial dynamics in clinical practice. Thus, significant challenges remain with respect to translating results from animal models to human applications.

The regulatory role of mitochondrial dynamics can be explored in conjunction with the overall homeostasis of the neurovascular unit (NVU). The NVU framework can be used to examine mitochondrial dynamics within the context of NVU homeostasis. Dexmedetomidine increases the phosphorylation level of serine at position 637 of Drp1 in endothelial cells, thereby mitigating the mitochondrial fragmentation induced by ischemic stimulation. This drug does not directly inhibit neuronal apoptosis and instead primarily maintains the integrity of the blood–brain barrier by protecting vascular endothelial mitochondria (Zhou et al. 2021). Thus, in addition to neuronal protection, vascular mitochondrial protection may serve as a secondary therapeutic target. Astrocytes can transport functional mitochondria to damaged neurons via nanotubes or exosomes, with related responses regulated through the AMPK/mTOR signaling pathway (Deng, Duan, et al. 2022; Lan et al. 2026). Nicotinamide riboside, a precursor of nicotinamide adenine dinucleotide (NAD), not only optimizes neuronal energy metabolism but also enhances astrocyte mitochondrial function, thereby amplifying the protective effects of intercellular mitochondrial transport (Deng, Duan, et al. 2022; Zhu et al. 2025). This type of intervention differs from traditional therapeutic approaches focused primarily on repairing damaged cellular structures; however, it remains in the preclinical research phase (Piao et al. 2025; Yang et al. 2022), and human trial data to validate its efficacy and safety in regulating the homeostasis of NVU s are lacking. Additionally, lifestyle interventions and natural bioactive compounds can exert synergistic effects through multiple pathways. Rhodiolin (Wen et al. 2022) and gastrodin (Chen, Yang, Wang, Chen, et al. 2024) alleviate vascular dementia‐associated mitochondrial dysfunction by regulating mitochondrial dynamics. Moreover, kaempferol inhibits mitochondrial fission and reduces hypoxia‐induced neuronal damage (Wu et al. 2017). Furthermore, lifestyle interventions such as exercise pretreatment increase the expression of Opa1, a protein associated with mitochondrial fusion, in rats following cerebral ischemia (Zhang et al. 2014). These results provide a basis for the application of exercise interventions in perioperative stroke prevention and control.

Future research could proceed in several directions. Specific antibodies and molecular probes targeting posttranslational modification sites of Drp1 could be developed to enable real‐time in vivo monitoring of relevant biomarkers. Additionally, single‐cell and spatial omics technologies could be used to elucidate the differences in the regulatory mechanisms of mitochondrial dynamics among various cell types within the NVU. Novel drugs targeting posttranslational modifications of RNA or proteins should be investigated, and their efficacy and mechanisms should be validated in nonhuman primate models of cerebral infarction. In summary, these research efforts are expected to bridge the gap between basic animal experiments and clinical applications.

### 6.4 Cerebral Ischemia–Reperfusion Injury

**FIGURE 7:**
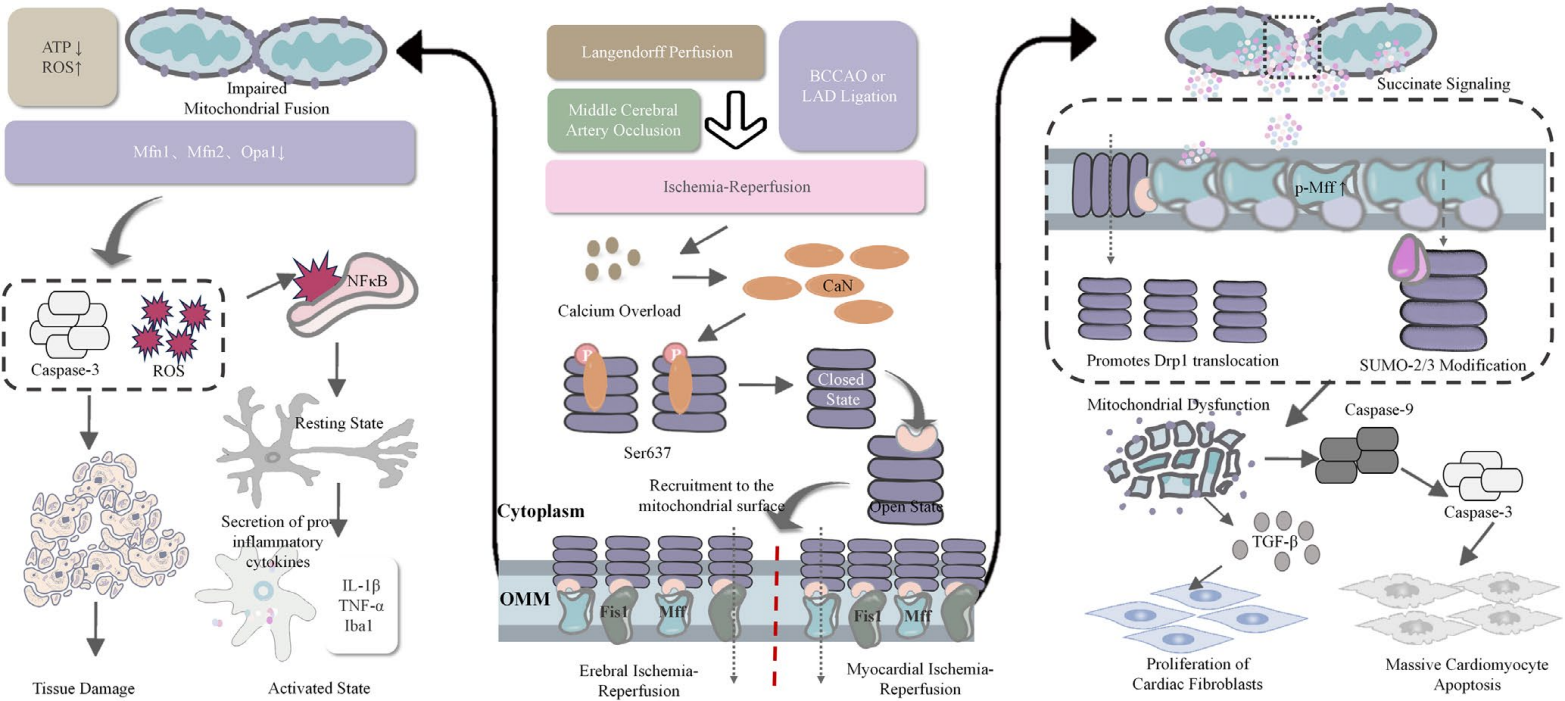
Schematic illustration of mitochondrial dynamics in cerebral and myocardial ischemia–reperfusion injury: shared mechanisms and organ‐specific differences. This schematic illustrates mitochondrial dynamics in cerebral and myocardial ischemia–reperfusion (I/R) injury, highlighting shared and distinct mechanisms. Both involve models such as Langendorff perfusion for myocardial, bilateral common carotid artery occlusion (BCCAO), and MCAO for cerebral. The shared mechanism is calcium overload activating calcineurin, triggering Drp1 translocation to the outer mitochondrial membrane via Fis1/Mff for excessive fission, with fusion proteins Mfn1, Mfn2, and Opa1 downregulated. For cerebral I/R on the left, this induces ROS, caspase activation, microglial polarization, and tissue damage. Myocardial I/R on the right features succinate signaling and SUMO2/3‐modified Drp1, driving mitochondrial dysfunction, caspase‐9/‐3‐mediated cardiomyocyte apoptosis, and cardiac fibroblast proliferation. It also emphasizes therapies like Drp1 inhibitor Mdivi‐1 and traditional Chinese medicine targeting mitochondrial dynamics to alleviate I/R injury in both organs.

I/R injury in the brain represents a severe complication following the restoration of blood flow after ischemic stroke. It constitutes a critical pathological pathway leading to irreversible neuronal death and neurological deficits. Mitochondria, which serve as cellular hubs for energy metabolism and signal integration, play central regulatory roles in this pathological process. In Figure 7, the regulatory similarities and differences in the dysregulation of mitochondrial dynamics between myocardial and cerebral tissues during I/R injury are systematically compared. Key changes in protein expression levels, organ‐specific phenotypes, and potential intervention strategies are shown to improve the understanding of cross‐organ commonalities and differences (Figure 7).

Compared with ischemia alone, I/R injury increases the expression of mitochondrial fission‐related protein 1 (Drp1) but significantly reduces the expression levels of fusion‐related proteins (Opa1) and mitochondrial fusion protein 2 (Mfn2) in MCAO model mice (Kumari et al. 2012). Compared with the sham‐operated group, the MCAO model group exhibited increased total Drp1 expression and significantly elevated levels of its active form, phosphorylated Drp1 (Ser616), ultimately resulting in an increased p‐Drp1 (Ser616)/Drp1 ratio, indicating increased mitochondrial fission activity (Liu, Su, et al. 2023). Additionally, studies have reported reduced cytoplasmic Drp1 expression and a corresponding increase in mitochondrial Drp1 expression in this model. This shift in the subcellular localization of Drp1 originates from the translocation of Drp1 to mitochondria, thereby promoting excessive mitochondrial fission (Liu, Su, et al. 2023). Concurrently, the expression of fission‐related factor 1 (Fis1) and Mff is increased in an MCAO model (Deng, Zhao, et al. 2022; Ge et al. 2024; Hu, Zeng, et al. 2020; Liu, Su, et al. 2023; Tang et al. 2023; Tang et al. 2020; Yang et al. 2020), whereas the expression of the fusion‐related proteins Mfn1, Mfn2, and Opa1 is significantly decreased (al Amir Dache and Thierry 2023; Chen, Duan, Zou, Yang, et al. 2024; Deng, Zhao, et al. 2022; Hu, Zeng, et al. 2020; Liu, Su, et al. 2023; Xu et al. 2025), confirming that Drp1‐mediated mitochondrial translocation is closely associated with an imbalanced state characterized by increased fission and reduced fusion. This finding aligns with the core pathological feature of the disruption of mitochondrial dynamics in cerebral I/R injury (Liu, Su, et al. 2023).

Posttranslational modifications of proteins involved in mitochondrial dynamics contribute to the process of cerebral I/R injury; this regulatory mechanism significantly differs across cell types and different stages of injury. Different phosphorylation sites on Drp1 can have distinct biological effects; in an MCAO model, phosphorylation at Ser616 has been shown to promote abnormal mitochondrial fission (Huang, Chen, et al. 2023). In specific neuronal subpopulations and during the early stages of hypoxic–ischemic injury, dephosphorylation at Ser637 promotes the translocation of Drp1 to mitochondria (Yuan et al. 2025). Upstream intracellular kinase pathways can regulate the changes in the levels of Drp1, thereby altering the functional state of the protein. In microglia, the E2F1/CDK5 signaling pathway regulates Drp1 phosphorylation, exacerbating mitochondrial structural abnormalities and intensifying neuroinflammatory responses (Yuan et al. 2025). In neurons, O‐GlcNAc transferases maintain the phosphorylation of Drp1 at Ser637, preventing abnormal protein localization and thereby preserving normal cellular physiological functions (Zhao et al. 2022). Different neuronal cell types have distinct regulatory pathways, which explains why mitochondria exhibit different morphological characteristics under similar injury conditions in various cell types. Cerebral I/R injury can also act on proteins related to mitochondrial dynamics through modifications such as ubiquitination and SUMOylation; these regulatory processes are closely linked to the mitochondrial quality control system. The metabolic fate of Mfn2 in brain tissue differs from that in cardiac muscle; in cerebral ischemia, this protein is primarily degraded via the ubiquitin–proteasome pathway. Injury stimuli promote the binding of the E3 ubiquitin ligase Mul1 to Mfn2, initiating the ubiquitination and degradation of Mfn2, which consequently inhibits mitochondrial outer membrane fusion (Huang, Li, et al. 2023). Posttranslational modifications of MFF can also participate in the regulation of mitochondrial morphology; AMPK‐mediated phosphorylation of MFF promotes SUMOylation at the Lys151 site, altering the structure of the complex formed by MiD49/51 and Drp1 and ultimately leading to changes in mitochondrial fragmentation (Seager et al. 2024). The imbalance in mitochondrial dynamics induced by cerebral I/R injury represents a systemic manifestation of functional compensation abnormalities arising from the inability of the mitochondrial quality control system to adapt to acute pathological stress.

In terms of intervention strategies, targeting posttranslational modifications specifically involves regulatory mechanisms that maintain normal mitochondrial dynamics in cells. The combination of astragaloside IV and chuanxiongzine alters the binding state of Drp1 with different SUMO subtypes; this combination weakens the interaction between Drp1 and SUMO1 while increasing the binding levels of Drp1 with SUMO2/3. Altering the pattern of protein SUMOylation can inhibit the mitochondrial fission process under pathological conditions, while the normal mitochondrial renewal process in cells remains unaffected (Chen, Yang, et al. 2024). Additionally, ginsenoside compound K can decrease Mul1 expression and block the ubiquitination and degradation of Mfn2, thereby restoring mitochondrial fusion function and improving cellular energy metabolism (Huang, Li, et al. 2023). Furthermore, the E2F1/CDK5/Drp1 pathway performs specific regulatory functions in microglia. Blocking this pathway via gene silencing or pharmacological strategies can mitigate mitochondrial damage induced by neuroinflammation while avoiding the adverse effects associated with the system‐wide inhibition of Drp1 activity (Yuan et al. 2025). Changes in mitochondrial dynamics are closely linked to cellular metabolism, mitophagy, and signaling pathways related to cell survival. AMPK connects the regulation of cellular metabolism and mitochondrial dynamics. Active compounds such as butylphthalide and ligustrin possess antioxidant capabilities and can activate the AMPK signaling pathway. These substances act on the AMPK/Mfn1 pathway to promote mitochondrial fusion (Zhu et al. 2024) and can also maintain the stability of mitochondrial dynamics via the AMPK/SIRT1/FoxO1 pathway (Tang et al. 2023). Under specific conditions, ligustrin can also induce moderate Drp1‐mediated mitochondrial fission through the AMPK signaling pathway, thereby initiating mitophagy to clear damaged organelles (Wu et al. 2022). The bidirectional regulation of mitochondrial fission and fusion can yield different therapeutic effects at various stages of disease progression. Melatonin and P2RY2 agonists can downregulate YAP phosphorylation levels and promote the transport of YAP into the nucleus. These substances can upregulate OPA1 expression or inhibit the mitochondrial fission process; mechanosensitive transcription factors have been shown to participate in the regulation of mitochondrial morphology (Nasoni et al. 2021; Xue et al. 2022). Compared with single‐target drugs, traditional Chinese medicine (TCM) formulations and natural single‐component compounds can act on multiple molecular targets, yielding superior overall regulatory effects. When a Danhong injection was applied in a model of hyperlipidemia combined with I/R injury, the expression of Drp1 and Mfn2 was regulated, the activity of respiratory chain complexes I to IV was restored, and mitochondria‐mediated apoptosis was inhibited. This drug improves mitochondrial morphology, structure, and physiological function and cell viability simultaneously (Du et al. 2023). Moreover, Naotai Formula regulates mitochondrial dynamics via Drp1‐related pathways while alleviating ferroptosis and suppressing inflammatory responses. This compound exerts synergistic regulatory effects on multiple modes of cell death (She et al. 2025). In addition to regulating the expression of mitochondrial‐related proteins, melatonin promotes mitochondrial transport via nanotunnels, thereby reducing the cGAS‐STING pathway‐mediated inflammatory response triggered by mtDNA release (Nasoni et al. 2021). This mechanism of action differs from traditional mitochondrial repair approaches and offers a new direction for the treatment of related diseases.

In summary, mitochondrial dysregulation in cerebral I/R injury manifests as excessive Drp1‐mediated fission and impaired Mfn2/Opa1 fusion and is finely regulated by posttranslational modifications such as phosphorylation and SUMOylation. Although these mechanisms have been thoroughly validated in animal models, the current evidence is derived almost entirely from preclinical studies, and no direct clinical trials have confirmed that targeting mitochondrial dynamics can improve outcomes in stroke patients. Although existing intervention strategies, such as the use of natural products or peptides, have demonstrated neuroprotective effects in rodents, their species specificity, blood–brain barrier penetration efficiency, and pharmacokinetic characteristics in humans remain unclear, limiting their clinical translation. In future research, validation studies using humanized models or early‐phase clinical trials should be prioritized, focusing on evaluating the safety margin, therapeutic window, and synergistic effects of selective modulators of Drp1 with reperfusion therapies to bridge the gap between basic research and clinical application.

Treatment strategies for age‐related brain diseases are summarized in Table 4.

**TABLE 4:** Treatment of aging‐related brain diseases.

Disease	Description	Intervention	Model	Effects on mitochondrial dynamics	Protein changes	References
Alzheimer's disease	Chinese medicine prescriptions and active extracts	Extract of Cynomorium songaricum (ECS)	HT22 cells treated with Aβ_25–35_	Changes mitochondrial dynamic	Drp1↓, p‐Drp1/Drp1↑	(Wang, Liu, et al. 2011)
		Tortoise plastron gelatin (TPG)	PC12 cells treated with Aβ_25–35_	Promotes fusion, inhibits fission	Drp1↓, p‐Drp1^S616^↓; Mfn1↑, Opa1↑	(Zhang et al. 2018)
		Deer antler gelatin (DAG)	PC12 cells treated with Aβ_25–35_	Inhibits fission	p‐Drp1^S616^↓, Fis1↓	(Zhang et al. 2018)
		Dengzhan Shengmai capsules (DZSM)	Administering intracerebroventricular injection of Aβ_42_ to the SD rats; primary neural cells treated with Aβ_42_	Promotes fusion, inhibits fission	p‐Drp1↓, Mff↓; Mfn1↑, Mfn2↑, Opa1↑	(Liu, Han, et al. 2024)
		*Cornus officinalis* Sieb. Et Zucc. (CoS)	Administering intracerebroventricular injection of Aβ_25–35_ to the C57BL/6J mice; PC12 cells treated with Aβ_25–35_	Promotes fusion, inhibits fission	Drp1↓, Fis1↓; Mfn1↑, Mfn2↑, Opa1↑	(Zacharioudakis and Gavathiotis 2023)
	Others	β‐lactolin	HT22 cells treated with Aβ_1–42_	Changes mitochondrial dynamic	Mfn2 mRNA↑	(Qipshidze‐Kelm et al. 2014)
		Thrombospondin‐1	HT22 cells treated with Aβ	Inhibits fission	p‐Drp1^S637^/Drp1↑	(Fu et al. 2021)
		Synthesis of diethyl (3,4‐dihydroxyphenethylamino) (quinolin‐4‐yl) methylphosphonate	SH‐SY5Y cells treated with Aβ_42_	Promotes fusion, inhibits fission	Drp1↓, Fis1↓; Mfn1↑	(Yang, Yu, et al. 2017)
		Cerium oxide nanoparticles	Primary cortical neurons treated with Aβ_25–35_	Inhibits fission	p‐Drp1^S616^/Drp1↓	(Li, Dang, et al. 2022)
Parkinson's disease	Selective inhibitor of Drp1	Mdivi‐1	C57BL/6 mice treated with MPTP; SH‐SY5Y cells treated with rotenone	Inhibits translocation of DRP1 to mitochondria	Cyto:Drp1↑, Mito:Drp1↓	(Su, Li, Wang, et al. 2023)
			SD rats treated with rotenone	Inhibits translocation of DRP1 to mitochondria	Cyto:Drp1↑, Mito:Drp1↓	(Shimizu et al. 2016)
	A mitochondrial fission inhibitor	FLZ	C57BL/6 mice treated with MPTP; SH‐SY5Y cells treated with MPP+	Inhibits fission	Drp1↓, Mito‐Drp1↓, p‐Drp1^S616^/Drp1↓	(Sharp et al. 2014)
	Inhibitor of Mitochondrial fission 1 protein (Fis1)/Drp1 interaction	P110	SH‐5YSY cells treated with MPP+	Inhibits fission	Drp1↓	(Zeng et al. 2020)
	Exercise	Treadmill training	C57BL/6 mice treated with MPTP	Inhibits fission	Mito‐Drp1↓	(Sun et al. 2016)
			SD rats treated with 6‐OHDA	Promotes fisson and fusion	Drp1↑; MFN2↑, L‐OPA1↑	(Byun et al. 2022)
	Electrical stimulation	Endurance exercise (EE)	C57BL/6 mice treated with MPTP	Promotes fusion, inhibits fission	p‐Drp1^s637^ ↑; Mfn2↑, Opa1↑	(Wang et al. 2017)
		Subthalamic nucleus deep brain stimulation	Monkey treated with MPTP	Promotes fusion, inhibits fission	Mito:Drp1↓; Opa1↑	(Li, Xu, et al. 2022)
	Chinese medicine prescriptions and active extracts	Anodal transcranial direct current stimulation	C57BL/6 mice treated with MPTP	Inhibits fission	Drp1↓	(Fan, Li, et al. 2022)
		Loganin	C57BL/6 mice treated with MPTP	Inhibits fission	Drp1↓	(Wang, Su, et al. 2011)
	Others	Thymoquinone	Wistar rats treated with rotenone	Inhibits fission	Drp1↓	(Preston et al. 2024)
	
		Andrographolide	C57BL/6 mice treated with MPTP; SH‐SY5Y cells treated with rotenone	Inhibits translocation of DRP1 to mitochondria	Cyto:Drp1↑, Mito:Drp1↓	(Su, Li, Wang, et al. 2023)
		Da‐Bu‐Yin‐Wan (DBYW)	SH‐SY5Y cells treated with MPP+	Promotes fusion, inhibits fission	Drp1↓, Fis1↓; Mfn1↑, Mfn2↑, Opa1↑	(He et al. 2019)
		Qian‐Zheng‐San (QZS)	SH‐SY5Y cells treated with MPP+	Promotes fusion, inhibits fission	Drp1↓, Fis1↓; Mfn1↑, Mfn2↑, Opa1↑	(He et al. 2019)
		Bu‐Yin‐Qian‐Zheng Formula	SH‐SY5Y cells treated with MPP+	Promotes fusion, inhibits fission	Drp1↓, Fis1↓; Mfn1↑, Mfn2↑, Opa1↑	(Ding et al. 2018)
		Carnosic acid	SH‐SY5Y cells treated with 6‐OHDA	Promotes fusion, inhibits fission	Fis1↓; Mfn2↑, Opa1↑	(Hao et al. 2024)
		Mangiferin	C57BL/6 mice treated with MPTP	Inhibits fission	Mito‐Drp1↓	(Ghahremani et al. 2018)
		Ferulic acid	Wistar rats treated with 6‐OHDA	Inhibits fission	Drp1↓	(Van Der Rijt et al. 2020)
		Irison	C57BL/6J mice treated with MPTP; SH‐SY5Y cells treated with MPP+/rotenone	Promotes fusion	Mice:Mfn1↑, Mfn2↑ Cell: p‐Drp1^S616^/Drp1↑; Mfn1↑, Mfn2↑, Opa1↑	(Li, Fan, et al. 2016)
		Melatonin	Primary rat cortical neuron treated with MPP+	Inhibits fission	Mito‐Drp1↓	(Quiroga et al. 2020)
	NAC (N‐acetylcysteine)	Wistar rats treated with rotenone	Inhibits fission	Drp1↓	(Li, Li, et al. 2020)
	Selective inhibitor of Drp1	TSA (Trichostatin A)	SH‐SY5Y cells treated with different doses of MPP+	Promotes fusion	Mfn1↑, Mfn2↑	(Ponce et al. 2020)
Water‐soluble coenzyme Q10		HT22 cells treated with Rotenone	Inhibits fission	p‐Drp1↓, Fis1↓; Mfn2↑	(Zhang, Zhang, et al. 2022)
	
		5‐HD (inhibit mitoKATP channels)	SD rats treated with rotenone; PC12 cells treated with rotenone	Mitochondrial dysfunction	Fis1↑; Mfn2↑, Opa1↑	(Spiegel et al. 2016)
		Uric acid	C57BL/6J mice treated with MPTP; primary cultured neural precursor cells treated with MPP+	Promotes fusion, inhibits fission	Drp1 tetramer ↓, Drp1 monomer↓; Mfn1↑, Mfn2↑, Opa1↑	(Hagenbuchner et al. 2018)
		Mdivi‐1	A four‐vessel occlusion (4‐VO) model in SD rats	Inhibits fission	Cyto: Drp1↑, Mito:Drp1↓	(Paillard et al. 2013)
Dexmedetomidine	Primary cortical neurons treated with OGD	Inhibits fission	p‐Drp1^S637^/Drp1↑	(Li, Yang, et al. 2022)
Cerebral infraction	A sedative	Kaempferol	Middle cerebral artery occlusion in SD rats (MCAO); human brain microvascular endothelial cells treated with OGD	Inhibits fission	Mito:p‐Drp1^S637^/Drp1↑	(Ooi et al. 2021)
			Primary cortical neurons treated with OGD	Inhibits fission	p‐Drp1^S637^/Drp1↑	(Li, Yang, et al. 2022)
	Chinese medicine prescriptions and active extracts	Gastrodin	Bilateral common carotid artery occlusion (BCCAO) in SD rats	Promotes fusion, inhibits fission	Drp1↓, Fis1↓; Mfn1↑, Mfn2↑	(Jin et al. 2011)
	Selective inhibitor of Drp1	Salvinorin A (SA)	Middle cerebral artery occlusion in SD rats (MCAO); human brain microvascular endothelial cells treated with OGD	Promotes fusion	Mfn2↑	(Qi et al. 2018)
	
		β‐asarone	Bilateral common carotid artery occlusion and reperfusion in ICR mice	Promotes fusion	Mfn2↑, Opa1↑	(Song, Gong, et al. 2015)
3‐n‐butylphthalide	PC12 cells treated with OGD	Promotes fusion, inhibits fission	Drp1↓, Fis1↓; Mfn1↑, Mfn2↑	(Zhang, Zhu, et al. 2024)
	
		Mdivi‐1	A four‐vessel occlusion and reperfusion (4‐VO) in Wistar rats	Inhibits translocation of DRP1 to mitochondria	p‐Drp1^S637^↑, Mito:Drp1↓	(Hou et al. 2018)
Danhong injection	Middle cerebral artery occlusion and reperfusion in SD rats (MCAO)	Inhibits fission	Cyto: Drp1↑, Mito:Drp1↓	(Shin et al. 2022)
Cerebral ischemia reperfusion injury	Chinese medicine prescriptions and active extracts	Atractylenolide III	Middle cerebral artery occlusion and reperfusion in SD rats (MCAO)	Promotes fusion, inhibits fission	Mito:Drp1↓, Fis1↓; Mfn1↑, Mfn2↑, Opa1↑	(Robert et al. 2021)
			Middle cerebral artery occlusion and reperfusion in C57BL/6J mice (MCAO); primary microglia cells treated with OGDR	Inhibits fission	Cyto: p‐Drp1^S616^/Drp1↑, Mito:Drp1↓	(Hall et al. 2021)
	Others	Ligustilide	HT22 cells treated with OGDR	Inhibits fission	Drp1↑, Fis1↑	(Palee et al. 2019)
		*Panax ginseng* and Angelica sinensis (CPA)	Middle cerebral artery occlusion and reperfusion in SD rats (MCAO)	Inhibits fission	Drp1↓	(Feng et al. 2019)
		Rehmapicroside	Middle cerebral artery occlusion and reperfusion in SD rats (MCAO)	Inhibits fission	Drp1↓	(Jin et al. 2018)
	
		Salidroside	Middle cerebral artery occlusion in C57BL/6J mice (MCAO); Primary cortical neurons were treated with OGD	Promotes fusion, inhibits fission	p‐Drp1/Drp1↓, Mff↓; Mfn1↑, Mfn2↑	(Su, Li, Shi, et al. 2023)
Cannabidiol	Middle cerebral artery occlusion and reperfusion in SD rats (MCAO); HT22 cells were treated with OGDR	Promotes fusion	Mfn2↑	(Li et al. 2023)
	
		Irisin	Middle cerebral artery occlusion and reperfusion in C57BL/6J mice (MCAO)	Promotes fusion, inhibits fission	Drp1↓, Fis1↓; Mfn2↑, Opa1↑	(Mushtaq et al. 2021)
		Astragaloside IV	Middle cerebral artery occlusion and reperfusion in SD rats (MCAO)	Promotes fusion, inhibits fission	Drp1↓, Fis1↓, Mff↓; Mfn1↑, Mfn2↑, Opa1↑	(Tokuyama et al. 2022)
		Ligustrazine	Middle cerebral artery occlusion and reperfusion in SD rats (MCAO)	Promotes fusion, inhibits fission	Drp1↓, Fis1↓, Mff↓; Mfn1↑, Mfn2↑, Opa1↑	(Tokuyama et al. 2022)
		Astragaloside IV combined with ligustrazine	Middle cerebral artery occlusion and reperfusion in SD rats (MCAO)	Promotes fusion, inhibits fission	Drp1↓, Fis1↓, Mff↓; Mfn1↑, Mfn2↑, Opa1↑	(Tokuyama et al. 2022)
20 (R)‐ginsenoside Rg3	Middle cerebral artery occlusion and reperfusion in SD rats (MCAO); PC12 cells treated with OGDR	Promotes fusion, inhibits fission	Drp1↓; Mfn2↑, Mfn1↑	(Guo et al. 2022)
	
		Dl‐3‐n‐butylphthalein	Middle cerebral artery occlusion and reperfusion in C57BL/6 mice (MCAO)	Promotes fusion	Mfn1↑, Mfn2↑, Opa1↑	(Chen, Ma, Song, Hua, et al. 2024)
		Phelligridimer A	HT22 cells treated with OGDR	Promotes fusion	Mfn2↑	(Hu, Guo, et al. 2022)
		Melatonin	HT22 mouse hippocampal neuroblastoma cells were treated with OGDR	Promotes fusion, inhibits fission	Drp1↓; MFN2	(Ishihara et al. 2015)
Cyclosporin	Left middle cerebral artery occlusion and reperfusion in SD rats (LMCA)	Inhibits fission	Drp1↓	(Scheckhuber et al. 2007)

		Cyclosporin +Melatonin	Left middle cerebral artery occlusion and reperfusion in SD rats (LMCA)	Inhibits fission	Drp1↓	(Scheckhuber et al. 2007)
			Left middle cerebral artery occlusion and reperfusion in SD rats (LMCA)	Inhibits fission	Drp1↓	(Scheckhuber et al. 2007)
		N‐acetyl‐L‐cysteine	Transient middle cerebral artery occlusion/reperfusion (tMCAO/R) Wistar rats	Inhibits translocation of Drp1 to mitochondria	Cyto:Drp1↑, Mito:Drp1↓	(Nunnari and Suomalainen 2012)
Volvalerenic acid A		Middle cerebral artery occlusion and reperfusion in SD rats (MCAO)	Promotes fusion, inhibits fission	Fis1↓; Mfn2↑, Opa1↑	(You et al. 2023)
	
		14,15—Epoxyeicosatrienoic acid	Middle cerebral artery occlusion and reperfusion in C57BL/6J mice (MCAO)	Promotes fusion, inhibits fission	Mfn1↑, Mfn2↑, Opa1↑, Fis1↓	(Nishimura et al. 2018)
		AG490 (an inhibitor of JAK2)	Middle cerebral artery occlusion and reperfusion in C57BL/6J mice (MCAO); primary microglia cells treated with OGDR	Inhibits fission	Cyto: p‐Drp1^S616^/Drp1↑, Mito:Drp1↓	(Hall et al. 2021)
		Arachidonyl‐2‐chloroethylamide (CB1 agonist)	Middle cerebral artery occlusion and reperfusion in SD rats (MCAO); HT22 cells treated with OGDR	Inhibits fission	Drp1↓, Fis1↓, Mff↓	(Ishikita et al. 2016)
		Meldonium	Primary hippocampal neurons treated with OGDR	Promotes fusion, inhibits fission	p‐Drp1^S637^↑; Mfn1↑, Mfn2↑, Opa1↑	(Palee et al. 2019)
Hypothermia	Middle cerebral artery occlusion and reperfusion in SD rats (MCAO)	Inhibits fission	Fis1↓	(Fan, Li, et al. 2022)

## 7 Mitochondrial Dynamics in Cardiocerebral Comorbidity

The high prevalence of cardiocerebral comorbidity in clinical practice suggests that systemic pathological conditions such as hypertension and diabetes may simultaneously damage the heart and brain through common molecular mechanisms, with mitochondrial dysregulation potentially serving as a central link connecting the damage to these two organs. In hypertension‐induced models of cardio‐cerebral comorbidity, the AngII–ROS–Drp1 axis is hyperactivated, leading to increased phosphorylation at the Ser616 site of Drp1 and decreased phosphorylation at the Ser637 site. This reduces the expression and function of Mfn2 and Opa1, resulting in mitochondrial network fragmentation, reduced ATP synthesis, and ROS bursts, which lead to the activation of the mitochondrial apoptosis pathway. Cardiac and brain tissues respond differently to this imbalance. Cardiomyocytes are highly sensitive to changes in energy supply; the loss of fusion capacity directly leads to contractile dysfunction and ventricular remodeling. In contrast, neurons rely on Mfn2 to maintain the anchoring of mitochondria to the ER and facilitate axonal transport. Animal models generated by different induction methods exhibit distinct dynamic phenotypes: toxin‐induced models are characterized by excessive Drp1‐mediated fission, whereas Mfn2 knockout models more directly reveal the consequences of fusion defects. In models of comorbidities such as hypertension combined with diabetes, abnormalities in cardiac and cerebral mitochondrial dynamics are more severe, and the response to single interventions is diminished, suggesting a synergistic amplification of damage. In terms of treatment, dapagliflozin, empagliflozin, metformin, melatonin, and certain traditional Chinese medicine compounds can simultaneously improve cardiac and cerebral mitochondrial dynamics; these agents modulate Drp1 phosphorylation and protect Mfn2/Opa1 function. Physical interventions such as exercise and cold exposure also demonstrate bidirectional regulatory potential. Therefore, analyzing cardiac and brain diseases within a unified framework of mitochondrial dynamics helps elucidate the molecular basis of comorbidities. The current evidence suggests that mitochondrial dysregulation may drive the progression of comorbidities. Future efforts should focus on the establishment of animal models of comorbidities that more closely mimic clinical reality and multicenter clinical trials targeting mitochondrial dynamics to validate the translational value of this framework. The key genetic intervention studies establishing causal links between mitochondrial dysregulation and cardio‐cerebral diseases are summarized in Table 5.

**TABLE 5:** Genetic interventions targeting mitochondrial dynamics.

Gene	Species/cell model	Intervention strategy	Disease model	Effect on mitochondrial dynamics	Phenotypic outcome (exacerbated/protective)	Causal relationship established	References
*Mfn1* + *Mfn2*	Mice (4–6 weeks old)	Cardiomyocyte‐specific double knockout	Acute myocardial infarction (LAD ligation followed by I/R)	Mitochondrial fragmentation, impaired baseline cardiac function	Significantly reduced infarct size (protective)	Yes (double knockout attenuates I/R injury)	(Hall et al. 2021)
*Mfn2*	Adult mice	Whole‐brain/hippocampal knockout (AAV‐Cre mediated)	No exogenous injury	Mitochondrial fragmentation, defective fusion	Neuronal death (exacerbated)	Yes (fusion deficiency directly drives neurodegeneration)	(Han et al. 2020)
*Mfn2*	Mice	Midbrain dopaminergic neuron‐specific knockout	No exogenous injury (mimicking Parkinson's disease)	Mitochondrial dysfunction and fragmentation	PD‐like symptoms and progressive neuronal loss (exacerbated)	Yes (*Mfn2* deficiency causes PD pathogenesis)	(Filograna et al. 2021)
*Mfn2*	Mice	Overexpression (AAV‐mediated)	MPTP‐induced Parkinson's disease model	Restored fusion equilibrium, reduced Drp1 mitochondrial translocation	Attenuated dopaminergic neuron degeneration (protective)	Yes (overexpression confers neuroprotection)	(Zhao et al. 2021)
*Opa1*	Primary neurons	Overexpression (plasmid transfection)	Aβ_1_₋_42_ challenge	Inhibited pathological mitochondrial fragmentation	Attenuated mitochondrial dysfunction and neuronal apoptosis (protective)	Yes (overexpression mitigates Aβ‐induced injury)	(Alikhanzade et al. 2025; Wang et al. 2008)
*MiD49* + *MiD51*	Mouse cardiomyocytes	Dual silencing (siRNA‐mediated)	Myocardial ischemia–reperfusion injury	Inhibited Drp1‐mediated excessive fission	Attenuated mPTP opening and reduced infarct size (protective)	Yes (dual silencing confers cardioprotection)	(Escobar‐Henriques and Anton 2013; Han et al. 2021)
*MTFP1*	Mouse cardiomyocytes	Overexpression (AAV9‐mediated)	Acute myocardial infarction	Promoted mitochondrial fusion (as a fusion cofactor)	Improved post‐infarction cardiac functional recovery (protective)	Yes (overexpression enhances cardiac repair)	(Hu et al. 2026)
*Siah2*	Mice (whole‐brain)	Constitutive gene knockout	Cerebral ischemia (oxygen–glucose deprivation/reperfusion)	Inhibited Drp1‐mediated pathological fission	Attenuated neuronal injury and improved neurological outcomes (protective)	Yes (knockout confers neuroprotection)	(Scortegagna et al. 2014; Sisalli et al. 2020)

## 8 Discussion

The heart and brain are the core organs of the body that consume substantial energy, and cardiovascular and cerebrovascular comorbidities are frequently observed in patients with diseases such as AS, which is particularly prevalent among elderly patients (Zhao et al. 2023). Recent multiorgan studies using colocalization analysis with magnetic resonance imaging and genetic data have confirmed a causal genetic relationship between adverse cardiac structural features, such as myocardial wall thickness, and stroke risk, although the specific molecular mechanisms remain unclear. An imbalance in mitochondrial dynamics may serve as a key molecular mediator of this genetic association. Recent research has indicated that excessive activation of the mitochondrial fission protein Drp1 accelerates vascular endothelial cell damage by promoting ROS production and apoptosis. This finding reveals a common pathway linking cardiac and cerebral injury in the progression of AS, which provides direct molecular evidence for the pathological association between cardiovascular and cerebrovascular diseases (Wang et al. 2017). This study is the first to systematically integrate three dimensions, namely, molecular regulation, disease‐specific manifestations, and cross‐organ synergistic interventions. An imbalance in mitochondrial dynamics is identified as a common core mechanism in degenerative diseases of the heart and brain. The crucial role of mitochondrial dynamics in maintaining the normal function of cardiomyocytes, neurons, and VSMCs is elucidated, and the limitations of traditional independent research on cardiovascular and cerebrovascular diseases are addressed. This provides a novel theoretical foundation for risk prediction and the comprehensive prevention and treatment of cardiocerebral comorbidities.

In cardiac aging and related diseases, dysfunction of mitochondrial dynamics is characterized by excessive Drp1‐mediated fission and insufficient Mfn2/Opa1‐mediated fusion (Chaanine et al. 2019; Ciocci Pardo et al. 2019; Ishikita et al. 2016). Specifically, under hypertensive conditions, ROS increase the activity of Drp1 through oxidative modification while accelerating Mfn2 ubiquitination and degradation, resulting in a vicious cycle that promotes fission and inhibits fusion (Minjares et al. 2023; Pemberton et al. 2025). During the progression of AS, ox‐LDL downregulates Opa1 and upregulates Drp1, thereby promoting the proliferation of VSMCs (Fang et al. 2022; Xie et al. 2020). During myocardial I/R injury, the synergistic acetylation and phosphorylation of Drp1 increase its mitochondrial translocation capacity, whereas AMPK inhibitors mitigate injury by reducing Drp1 acetylation (Zhang, Wang, et al. 2024). However, mitochondrial fission and fusion are not unidirectional independent processes but rather existing in a dynamic equilibrium that is jointly regulated by the cellular metabolic state, stress signals, and inflammatory factors. A high‐glucose environment increases the sensitivity of cardiomyocytes to I/R injury, which is associated with elevated cytoplasmic mtDNA levels and the increases in the S616 phosphorylation levels of Drp1 (Dubois et al. 2024). Conversely, AMPK activation or SIRT3 upregulation increases Mfn2 expression, restores the integrity of the mitochondrial network, and thereby mitigates myocardial injury (Finocchiaro et al. 2024). This bidirectional regulatory mechanism reveals that clinical interventions must balance the inhibition of excessive fission with the restoration of fusion capacity rather than targeting a single pathway. The interventions summarized in this study, including Mdivi‐1, irbesartan, and aerobic exercise, all exert cardioprotective effects by correcting the aforementioned imbalance (Chen, Song, and Yao 2022; Ishikita et al. 2016; Jiang et al. 2014). These findings directly validate the feasibility of using mitochondrial dynamics as a therapeutic target.

In neurological disorders, the dysregulation of mitochondrial dynamics involves the bidirectional regulation of core pathological features such as Aβ accumulation and α‐Syn aggregation, with defects in mitochondrial fusion serving as key drivers of neuronal injury. Significant decreases in Mfn1/2 and Opa1 expression levels are observed in both AD and PD models, leading to mitochondrial network fragmentation, disruption of energy supplies, and disruption of calcium homeostasis, ultimately accelerating neuronal death. In the brain tissue of AD patients, excessive phosphorylation of the S616 site of Drp1 via the CDK5/GSK3β signaling pathway amplifies fission signals and induces mitochondrial fragmentation (Baek et al. 2017). In the accelerated aging SAMP8 mouse model of AD, Drp1, Mfn2, and Opa1 expression was reduced in both cortical and hippocampal regions, indicating the concurrent impairment of fission and fusion functions. In Parkinson's disease models, MPTP induces mitochondrial translocation of Drp1 and decreases Mfn2 expression, whereas the overexpression of Mfn2 or the inhibition of Drp1 activity significantly reverses the loss of dopaminergic neurons (Feng et al. 2020; Zhao et al. 2021). In experimental autoimmune encephalomyelitis (EAE) mice, mitochondrial fission and fusion pathways are both impaired, confirming the prevalence of fusion defects in neurodegenerative diseases. These studies suggest that restoring mitochondrial fusion may be a more effective neuroprotective strategy than simply inhibiting fission.

Through a systematic analysis of the pathological mechanisms underlying age‐related cardiovascular and cerebrovascular diseases and an investigation of key molecular pathways, we clarified that imbalances in mitochondrial dynamic homeostasis are central to mediating the progression of age‐related diseases through infection, metabolic abnormalities, and stress responses. For instance, *Porphyromonas gingivalis* simultaneously promotes AS and neurotoxicity by inducing phosphorylation at the S616 site of Drp1 as well as its mitochondrial translocation (Feng et al. 2020; Xu et al. 2021), whereas Mdivi‐1 reverses this pathological process, offering a novel perspective for preventing cardiovascular and cerebrovascular diseases through oral health management. Early‐life stress disrupts synaptic mitochondrial function and lipid metabolism, which shares common pathogenic mechanisms with amyloid deposition and ultimately increases the risk of AD (Butterfield and Halliwell 2019). Dysfunction of glucose metabolism, as an upstream event in AD (Butterfield and Halliwell 2019), leads to elevated p‐Tau levels, which can be reversed by L‐carnitine through improved mitochondrial function (Magi et al. 2021). These findings link seemingly unrelated diseases via mechanisms associated with mitochondrial dynamics, thereby expanding the framework for preventing and managing age‐related diseases.

However, existing research must be further refined, particularly in terms of animal model validation and clinical translation. First, the relative importance of excessive mitochondrial fission versus insufficient fusion across different diseases remains unclear. For instance, in heart failure models, some studies have shown decreased Opa1 expression and increased Drp1 expression, whereas other studies have reported reduced Drp1 expression. Compared with single‐defect mice, multigene knockout models of cardiovascular–cerebral comorbidities exhibit higher short‐term survival rates but develop pathological myocardial hypertrophy because of mitochondrial accumulation over time (Ordog et al. 2021). These findings suggest that the balance between fission and fusion may be more critical than either process alone; however, this finding must be validated in larger clinical trials and standardized animal models. Second, the disease‐specific regulatory mechanisms of posttranslational modification networks for mitochondrial dynamics‐related proteins must be further investigated. For instance, the distinct roles of the S616/S637 phosphorylation balance of Drp1 in cardiac and neurological diseases remain unexplored. Additionally, clinical translation faces three core challenges: the lack of standardized biomarkers, discrepancies between animal models and human diseases, and insufficient drug targeting specificity. To distinguish correlative observations from causal mechanisms, we compiled genetic intervention studies (knockout, knockdown, or overexpression) of key components associated with mitochondrial dynamics in cardio‐cerebral disease models. The interactions between mitochondrial dynamics and metabolic, inflammatory, and immune networks remain incompletely understood, and systems biology analyses are needed to elucidate multisystem regulatory patterns.

In summary, in this study, we systematically reviewed the regulatory mechanisms and intervention strategies associated with mitochondrial dynamics in age‐related cardiovascular and cerebrovascular diseases, with the aim of improving our understanding of the pathophysiology of cardiovascular–cerebrovascular comorbidities and clarifying the clinical value of mitochondrial dynamics‐related proteins as therapeutic targets. Future research should focus on five key directions centered on mitochondrial dynamics to develop prevention and treatment strategies for cardiovascular–cerebrovascular comorbidities. First, highly sensitive imaging techniques or blood biomarkers capable of dynamically monitoring mitochondrial function, such as circulating mitochondrial‐derived vesicles, must be developed to enable early risk assessment for cardiovascular and cerebrovascular diseases. With the use of these monitoring tools, humanized animal models of cardiovascular–cerebrovascular comorbidities should be established. These models should incorporate CRISPR technology to precisely mimic clinical mutations, thereby enabling evaluations of the long‐term safety of methods that regulate mitochondrial fission and fusion. Additionally, drugs capable of “bidirectional regulation” of mitochondrial dynamics, such as AMPK activators combined with SIRT3 upregulators, should be developed to simultaneously suppress excessive fission and restore fusion capacity, thereby increasing the specificity of these interventions. Furthermore, to refine therapeutic guidance, single‐cell multiomics technologies should be employed to elucidate mitochondrial network remodeling characteristics in cardiomyocytes and neurons across different pathological stages, providing stratified evidence for precision interventions. Finally, mitochondrial dynamics should be integrated with genetic, metabolic, and imaging data to develop comprehensive risk models for cardiovascular and cerebrovascular diseases, which could advance the widespread application of precision medicine techniques in aging populations. Through these systematic explorations, we can effectively prevent and treat cardiovascular–cerebrovascular comorbidities in the future, thereby reducing the disease burden in aging societies and providing robust scientific evidence.

## Author Contributions

**Yuyao Yin:** conceptualization, literature collection, writing – original draft, visualization, investigation of literature sources, validation of key mechanisms, figure revision. **Zijian Li:** methodology, data analysis, writing – review and editing, supervision, funding acquisition, project administration. All authors have read and agreed to the published version of the manuscript.

## Funding

This work was supported by the Science and Technology Program Joint Program of Liaoning Province (Project of Fundamental Research for Application), No. 2023JH2/101700204.

## Conflicts of Interest

The authors declare no conflicts of interest.

## Data Availability

Data sharing not applicable to this article as no datasets were generated or analysed during the current study.
